# Exploring the Biomedical Potential of Terpenoid Alkaloids: Sources, Structures, and Activities

**DOI:** 10.3390/molecules29091968

**Published:** 2024-04-25

**Authors:** Xuyan Wang, Jianzeng Xin, Lili Sun, Yupei Sun, Yaxi Xu, Feng Zhao, Changshan Niu, Sheng Liu

**Affiliations:** 1 School of Pharmacy, Yantai University, Yantai 264005, China; 17851072916@163.com (X.W.); syp6935@163.com (Y.S.); unicy81@163.com (Y.X.); 2 School of Life Sciences, Yantai University, Yantai 264005, China; jianzeng77@sina.com; 3 College of Pharmacy, University of Utah, Salt Lake City, UT 84108, USA; lili.sun1989@gmail.com

**Keywords:** terpenoid alkaloids, natural products, biological activity

## Abstract

Terpenoid alkaloids are recognized as a class of compounds with limited numbers but potent biological activities, primarily derived from plants, with a minor proportion originating from animals and microorganisms. These alkaloids are synthesized from the same prenyl unit that forms the terpene skeleton, with the nitrogen atom introduced through β-aminoethanol, ethylamine, or methylamine, leading to a range of complex and diverse structures. Based on their skeleton type, they can be categorized into monoterpenes, sesquiterpenes, diterpenes, and triterpene alkaloids. To date, 289 natural terpenoid alkaloids, excluding triterpene alkaloids, have been identified in studies published between 2019 and 2024. These compounds demonstrate a spectrum of biological activities, including anti-inflammatory, antitumor, antibacterial, analgesic, and cardioprotective effects, making them promising candidates for further development. This review provides an overview of the sources, chemical structures, and biological activities of natural terpenoid alkaloids, serving as a reference for future research and applications in this area.

## 1. Introduction

Alkaloids, a diverse class of secondary metabolites, are widely distributed in nature, with more than 27,000 species identified to date, predominantly originating from the plant kingdom, though relatively few are found in the animal and microbial kingdoms [1]. They typically exhibit strong biological activities, including antitumor, antibacterial, insecticidal, and analgesic effects [2,3,4]. Among the numerous classes of alkaloids, terpenoid alkaloids (TeAs) occupy a pivotal position. These alkaloids are formed from terpenoids through amination reactions, making them aminated terpenes [5]. TeAs are classified as pseudo alkaloids primarily because their biosynthetic origins do not involve the amino acid pathway. Instead, terpenoid moieties in TeAs are biosynthesized from isoprene through the methylerythritol phosphate (MEP) pathway, while nitrogen atoms are typically introduced into the structures of terpenoids in the form of *β*-aminoethanol, ethylamine, or methylamine [1].

Despite the vast variety of alkaloids and terpenoids isolated from nature, only a tiny proportion of them conform to the structural features of TeAs. TeAs, as a natural product with diverse structures, are primarily divided into monoterpene, sesquiterpene, diterpene, and triterpene alkaloids according to the differences in their skeletons [6]. Among them, monoterpene alkaloids are derived from iridoid compounds, mainly concentrated in the plants of Bignoniaceae, Lamiaceae, Gentianaceae, and Scrophularia [7]. Sesquiterpene alkaloids are the least abundant class of TeAs, which are narrowly distributed in the plant kingdom and mainly concentrated in plants such as Dendrobium [5]. Diterpenoid alkaloids (DAs) are the most complex and numerous compounds in TeAs, mainly concentrated in the *Aconitum* and *Delphinium* plants of Ranunculaceae [8]. In addition, marine sponges are also an important source of diterpenoid alkaloids.

Although small in number, these alkaloids are widely bioactive. For example, incarvillateine, a monoterpene alkaloid with strong analgesic activity, isolated from the traditional Chinese medicine *Incarvillae sinensis* LAM., has become a significant lead compound in the development of new non-narcotic pain medications [9]. DAs have been used for many years as traditional medicines in China, Japan, Russia, Mongolia, and India [10]. Because of their severe toxicity, in ancient times, *Aconitum* roots were often used to hydrolyze highly toxic DAs (e.g., aconitine) into less toxic derivatives (e.g., benzylaconine) by soaking, boiling, or other processing methods [8,11]. Modern pharmacological studies have shown that diterpene alkaloids have significant anti-inflammation, analgesia, anticancer, and anti-arrhythmia effects [8]. Moreover, as a diterpenoid alkaloid, Crassicauline A has been clinically utilized as an anti-arrhythmic drug [12]. Similarly, cyclovirobuxine-D, a triterpene alkaloid, is also used clinically as an antiarrhythmic drug [13] and has been recognized as a lead compound for innovative analgesics [14].

The research significance and medical value of TeAs as a class of natural products with unique structures and a wide range of biological activities are clear. Given the complexity and variability of triterpenoid alkaloids’ structures and the constraints of space, this paper will focus on the sources, chemical structures, and biological activities of natural TeAs, excluding triterpene alkaloids, discovered in the past five years, hoping to provide a reference for the further research and application of TeAs.

## 2. Classes of Terpenoidal Alkaloids

### 2.1. Monoterpenoid Alkaloids

**Figure 1:**
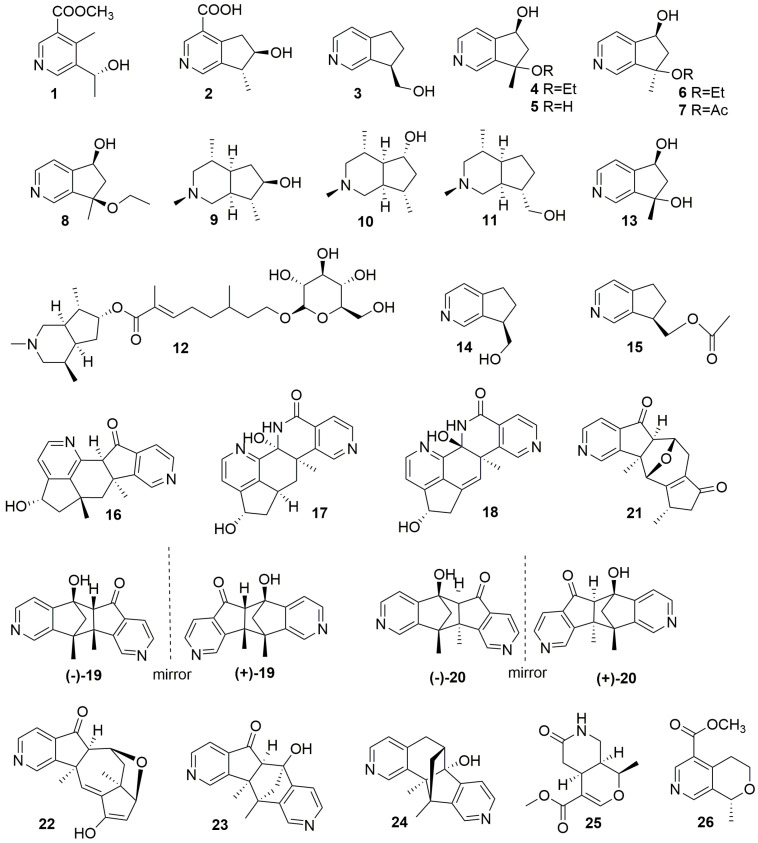
Structures of monoterpene alkaloids (**1**–**26**).

Monoterpenoid alkaloids represent a distinct class of alkaloids derived from iridoid glycosides, typically originating from loganin and secologanin after amination. According to Wang’s classification of monoterpene alkaloids, they can be divided into two categories: iridoids and secoiridoids [15]. This section discusses 26 monoterpenoid alkaloids isolated from plants, including 24 iridoid-type alkaloids (**1**–**24**) and two secoiridoid-type alkaloids (**25**–**26**). Specific plant sources are listed in Table 1. The chemical structure details are shown in Figure 1.

#### 2.1.1. Iridoid-Type Alkaloids (**1**–**24**)

The biosynthetic precursors of these alkaloids are iridoid glycosides. Based on the level of hydrogenation within their nitrogen-containing six-membered rings, they can be classified into four subtypes: pyridine ring type, piperidine ring type, dihydropyridine ring type, and tetrahydropyridine ring type [15].

Alstochonines A (**1**) and B (**2**) were isolated from the branches of *Alstonia scholaris* (Apocynaceae). Alstochonine A (**1**) was the first reported C-4 methylated nor-monoterpenoid alkaloid. Alstochonine B (**2**), processing a cyclopentyl[c]pyridine skeleton, is believed to be biosynthesized from iridotrial by ammonification, aromatization, and oxidation reactions [16].

(R)-10-hydroxyl-4-noractinidine (**3**) was extracted and isolated from *Rauvolfia vomitoria*’s trunk, the first reported monoterpene alkaloid in *R. vomitoria* (Apocynaceae) [17].

Delavatines C-E (**9**–**11**), three monoterpene alkaloids with a cyclopentane[c]piperidine skeleton, were isolated from whole plants of *Incarvillea delavayi* (Bignoniaceae) [18].

Incarvine G (**12**), a novel monoterpene alkaloid isolated from *Incarvillea sinensis* Lam., is an ester composed of a monoterpene alkaloid with a cyclopentane[c]piperidine skeleton and glucose [19].

Isoxerine (**13**), isolated from the roots of *Scrophularia ningpoensis*, was named due to its absolute configuration of C-7 being 7S, differing from oxerine [20].

Forsyqinlingines C (**14**) and D (**15**) were isolated from the ripe fruits of *Forsythia suspensa* (Oleaceae), with the structures determined by analysis of spectra including HR-ESI and NMR. Both alkaloids belong to a rare class of planar C9-monoterpenoid alkaloids [21].

The dimeric monoterpene alkaloids (±)-Caryopterisines A (**19**) and B (**20**) were identified as racemates isolated from *Caryopteris glutinosa* Rehder (Lamiaceae), and their absolute configurations were determined using calculated ECD spectra and X-ray diffraction analysis. They are two novel dimers with a 6/5/5/5/6 pentacyclic system. In addition, they can be biosynthesized by oxerine dehydration or oxygenation and subsequent Diels–Alder reactions [22].

Caryopterisines F-I (**4**–**7**) and caryopterisines C-E (**16**–**18**) were subsequently isolated from *C. glutinosa* Rehder. Caryopterisines F-I (**4**–**7**) are four monoterpene alkaloids containing the cyclopentyl[c]pyridine skeleton, while caryopterisines C-E (**16**–**18**) represent three novel dimeric monoterpene alkaloids and are believed to be biosynthesized via the Diels–Alder reaction followed by aromatization rearrangement and a series of subsequent reactions [23]. Of these, caryopterisine C (**16**) has an unprecedented 6/5/6/6/5 pentacyclic ring framework, while caryopterisines D (**17**) and E (**18**) both have 6/6/6/5 fused ring frameworks.

Compounds **8** and **21**–**24** were isolated from the aerial parts of *Caryopteris mongolica* Bunge (Lamiaceae), a medicinal plant in Mongolia. Among them, (5S*,7R*)-7-Ethoxy-6, 7-dihydro-7-methyl-5*H*-cyclopenta[c]pyridin-5-ol (**8**) is a monoterpene alkaloid with a cyclopenta[c]pyridine framework [24]. (5aR*,6S*,10S*,11R*,11aR*)-10,11a-Dimethyl-6,7,9,10,11,11a-hexahydro-5*H*-6,11-epoxycyclopenta [6,7]azuleno [1,2-*c*]pyridin-5,8(5a*H*)-dione(**21**) and (5aR*,6S*,7aR*,8S*,11aR*)-10-Hydroxy-7a,11a-dimethyl-5a,6,7,7a,8,11a-hexahydro-5*H*-6,8-epoxycyclopenta [6,7]azuleno [1,2-*c*] pyridin-5-one(**22**) could be formed by fusion of the cyclopenta[c]pyridine and 4-Demethyliridoid, and (5R*,5aR*,10bS*,11R*)-5-Hydroxy-10b,11-dimethyl-5,5a,10b,11-tetrahydro-6*H*-5,11-methanopyrido [3′,4′:3,4]cyclopenta [1,2-*g*]isoquinolin-6-one(**23**) and (6S*,6aR*,11R*,11aS*)-6a-Hydroxy-11,11a-dimethyl-6,6a,11,11a-tetrahydro-5*H*-6,11-methanopyrido [3′,4′:4,5]cyclopenta [1,2-*h*]isoquinolin-5-one(**24**) are dimerization products of two cyclopenta[c]pyridine [24].

**Table 1:** Names and plant sources of monoterpene alkaloids (**1**–**26**).

No.	Compound Names	Sources	Plant Parts	Ref.
**1**	Alstochonine A	*Alstonia scholaris*	branch	[16]
**2**	Alstochonine B	*Alstonia scholaris*	branch	[16]
**3**	(R)-10-hydroxyl-4-noractinidine	*Rauvolfia vomitoria*	trunk	[17]
**4**	Caryopterisine F	*Caryopteris glutinosa*	whole plant	[23]
**5**	Caryopterisine G	*Caryopteris glutinosa*	whole plant	[23]
**6**	Caryopterisine H	*Caryopteris glutinosa*	whole plant	[23]
**7**	Caryopterisine I	*Caryopteris glutinosa*	whole plant	[23]
**8**	(5S*,7R*)-7-Ethoxy-6,7-dihydro-7-methyl-5*H*-cyclopenta[c]pyridin-5-ol.	*Caryopteris mongolica* Bunge	aerial part	[24]
**9**	Delavatine C	*Incarvillea delavayi*	whole plant	[18]
**10**	Delavatine D	*Incarvillea delavayi*	whole plant	[18]
**11**	Delavatine E	*Incarvillea delavayi*	whole plant	[18]
**12**	Incarvine G	*Incarvillea sinensis*	whole herb	[19]
**13**	Isoxerine	*Scrophularia ningpoensis*	root	[20]
**14**	Forsyqinlingine C	*Forsythia suspensa*	fruit	[21]
**15**	Forsyqinlingine D	*Forsythia suspensa*	fruit	[21]
**16**	Caryopterisine C	*Caryopteris glutinosa*	whole plant	[23]
**17**	Caryopterisine D	*Caryopteris glutinosa*	whole plant	[23]
**18**	Caryopterisine E	*Caryopteris glutinosa*	whole plant	[23]
**19**	(±)-Caryopterisine A	*Caryopteris glutinosa*	whole plant	[22]
**20**	(±)-Caryopterisine B	*Caryopteris glutinosa*	whole plant	[22]
**21**	(5aR*,6S*,10S*,11R*,11aR*)-10,11a-Dimethyl-6,7,9,10,11,11a-hexahydro-5*H*-6,11-epoxycyclopenta [6,7]azuleno [1,2-*c*]pyridin-5,8(5a*H*)-dione.	*Caryopteris mongolica* Bunge	aerial part	[24]
**22**	(5aR*,6S*,7aR*,8S*,11aR*)-10-Hydroxy-7a,11a-dimethyl-5a,6,7,7a,8,11a-hexahydro-5*H*-6,8-epoxycyclopenta [6,7]azuleno [1,2-*c*] pyridin-5-one.	*Caryopteris mongolica* Bunge	aerial part	[24]
**23**	(5R*,5aR*,10bS*,11R*)-5-Hydroxy-10b,11-dimethyl-5,5a,10b,11-tetrahydro-6*H*-5,11-methanopyrido [3′,4′:3,4]cyclopenta [1,2-*g*]isoquinolin-6-one.	*Caryopteris mongolica* Bunge	aerial part	[24]
**24**	(6S*,6aR*,11R*,11aS*)-6a-Hydroxy-11,11a-dimethyl-6,6a,11,11a-tetrahydro-5*H*-6,11-methanopyrido [3′,4′:4,5]cyclopenta [1,2-*h*]isoquinolin-5-one	*Caryopteris mongolica* Bunge	aerial part	[24]
**25**	Longiflorine	*Uncaria longiflora* var. *pteropoda*	leaf	[25]
**26**	Lomatogonin C	*Lomatogonium carinthiacum*	whole plant	[26]

#### 2.1.2. Secoiridoid-Type Alkaloids (**25**–**26**)

This kind of alkaloid is mainly distributed in Gentianaceae plants and derived from secoiridoid glycosides [15]. Longiflorine (**25**), isolated from the leaves of *Uncaria longiflora* var. *Pteropoda* (Rubiaceae), is a monoterpenoid alkaloid with a lactam ring derived from secologanin [26]. Lomatogonin C (**26**), isolated from dried whole plants of *Lomatogonium carinthiacum* (Gentianaceae), is a natural monoterpene alkaloid derived from secoiridoid [25].

### 2.2. Sesquiterpene Alkaloids (***27**–**32***)

**Figure 2:**
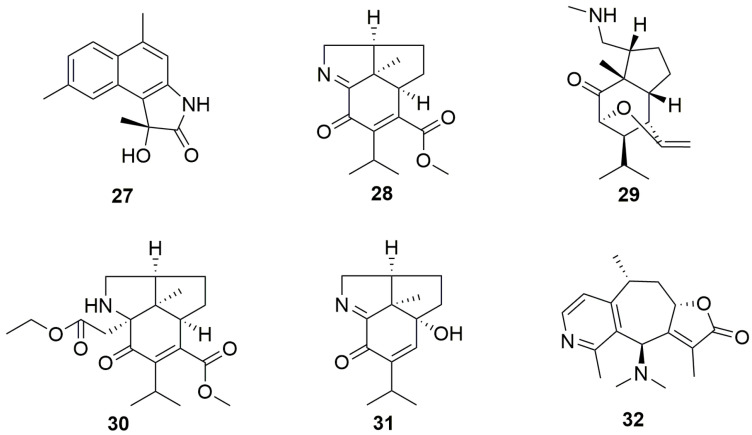
Structures of sesquiterpene alkaloids (**27**–**32**).

Sesquiterpene alkaloids represent the least abundant class of TeAs derived from sesquiterpenes and incorporate nitrogen atoms in the basic skeleton of sesquiterpenes [27]. This subsection mainly describes six sesquiterpene alkaloids from nature, including a rare alkaloid from the ocean. The plant sources are listed in Table 2, and the chemical structure details are shown in Figure 2.

**Table 2:** Names and plant sources of sesquiterpene alkaloids (**27**–**32**).

No.	Compound Name	Sources	Plant Parts	Ref.
**27**	Commipholactam A	*Resina Commiphora*	myrrh	[28]
**28**	Dendrofindline A	*Dendrobium findlayanum*	stem	[29]
**29**	Dendrofindline B	*Dendrobium findlayanum*	stem	[29]
**30**	Findlayine D	*Dendrobium findlayanum*	stem	[30]
**31**	Findlayine F	*Dendrobium findlayanum*	stem	[30]
**32**	Echinoflorine	gorgonian *Echinogorgia flora*	/	[31]

/: did not report.

Commipholactam A (**27**) was isolated from the dried myrrh of *Resina Commiphora* and represented a rare cadinane-type sesquiterpenoid. Unlike typical cadinane sesquiterpenoids, where ring C is usually present as a lactone, compound **27** appears as a lactam ring [28].

Dendrofindlines A-B (**28**–**29**) and Findlayines D-F (**30**–**31**) were all obtained from the dried stems of *Dendrobium findlayanum* and belong to the dendrobine-type alkaloid group [29,30]. Notably, Dendrofindline A (**28**) and Findlayine D (**30**) belong to the *seco*-lactone dendrobine-type alkaloid group. Findlayine D (**30**) is the first reported dendrobine-type alkaloid to feature a 2-ethoxy-2-oxoethyl group attached at C-2. Moreover, Dendrofindline B(**29**) is identified as a dendrobine-type alkaloid with a nitrogen-containing ring cleavage [29]. Findlayine F(**31**) is a nor-dendrobine-type alkaloid with a 5-decarboxylated structure [30].

Echinoflorine (**32**), isolated from the Gorgonian *Echinogorgia flora*, is a guaipyridine-type alkaloid with a *γ*-lactone-cyclohepta[c]pyridine skeleton, which is different from the cyclohepta[b]pyridine skeleton derived from terrestrial organisms [31].

### 2.3. Diterpenoid Alkaloids (DAs) (***33**–**289***)

DAs are the most abundant and structurally complex TeAs, characterized by numerous stereocenters. They typically originate from the amination of tetracyclic or pentacyclic diterpenes, forming heterocyclic systems possessing *β*-aminoethanol, methylamine, or ethylamine nitrogen atoms [32]. Based on the number of carbon atoms in the skeleton of DAs, they can be classified into three major categories: C18, C19, and C20 [33]. Shen Yong comprehensively reviewed the classification of diterpenoid alkaloids in 2020 [8]. Consequently, this article will not delve into an extensive discussion of this classification but will focus only on the classification of new members discovered in the past five years. This section describes 257 newly discovered natural DAs, including 11 C18-DAs, 139 C19-DAs, 84 C20-DAs, 14 Bis-DAs, and 9 other types of DAs. These DAs were predominantly isolated from the plants in the *Aconitum* and *Delphinium* genera, with only two new DAs isolated from the ripe fruits of *Forsythia suspensa*. Additionally, five new DAs were obtained from microorganisms and marine animals.

#### 2.3.1. C18-DAs (**33**–**43**)

**Figure 3:**
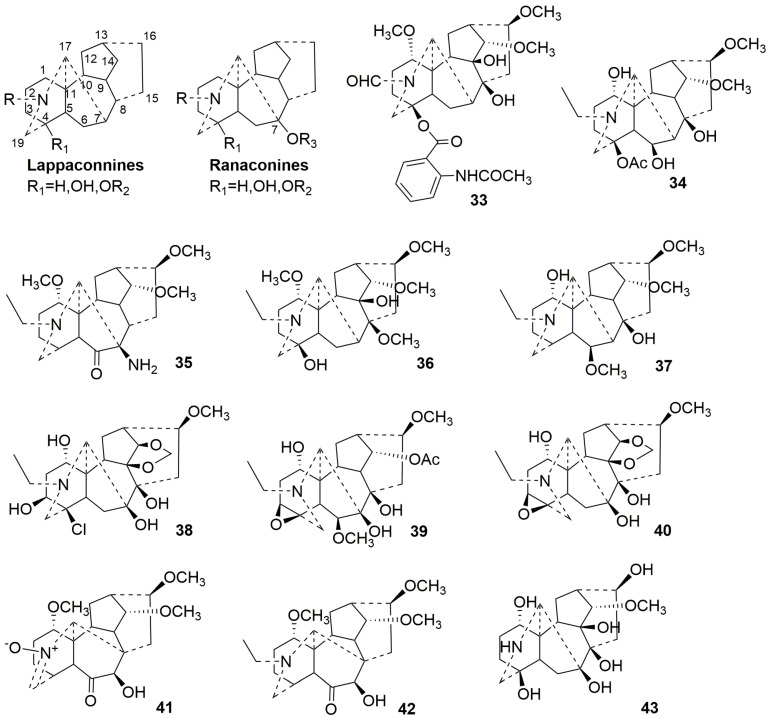
Structures of C18-DAs (**33**–**43**).

Without C18 in the structure, these alkaloids predominantly feature a 4-OH or ester substitution, with a few compounds having 3,4-epoxy substitution. According to the presence or absence of oxygen-containing groups at C7, they are classified into lappaconitines and ranaconitines [32]. Eleven C18-DAs (**33**–**43**) are described in this subsection. Plant sources are shown in Table 3. Detailed chemical structures are shown in Figure 3.

Compounds **33**–**37** are identified as lappaconine-type alkaloids, with four originating from *Aconitum* and only Naviconine (**33**) derived from *Delphinium* [34,35,36]. Amino groups are generally uncommon in DAs, whereas Leucostosine C(**35**) is the first naturally occurring DA to feature an amino group attached at C-7 [36].

Compounds **38**–**40**, obtained from *Aconitum,* are classified as ranaconitine-type DAs. Kirisine A (**40**) and B (**38**) are rare DAs with 9,14-methylenedioxy group. In addition, kirisine B (**38**), containing a chlorine substituent at C-4, represents a fourth example of DAs with a chlorine substituent [35].

Compounds **41** and **42** are rearranged C18-DAs, where the C7-C17 bond was rearranged to a C8-C17 bond [37]. Compounds **41** and **42** are derived from the rearrangement of ranaconitine-type DAs, and both contain an oxygen-containing hydroxyl group at C7. Additionally, Barpubenine A (**41**) is the first reported N-oxide in C18-DAs [37].

1-N-deethyl-1,16-demethoxy-1,16-dihydroxypyranaconidine(**43**) is a C18-nor-diterpene alkaloid with a skeleton similar to ranaconidine except that it lacks an N-ethyl group [38]. Furthermore, a hydroxyl group exists at C-16 instead of a methoxy group, which is common in C18-DAs [38].

**Table 3:** Names and plant sources of C18-DAs (**33**–**43**).

No.	Compound Name	Sources	Plant Parts	Ref.
**33**	Naviconine	*Delphinium naviculare* var. *lasiocarpum*	whole plant	[34]
**34**	Kirisine E	*Aconitum *kirinense** Nakai	root	[35]
**35**	Leucostosine C	*Aconitum leucostomum* Worosch	root	[36]
**36**	Leucostosine D	*Aconitum leucostomum* Worosch	root	[36]
**37**	Kirisine D	*Aconitum *kirinense** Nakai	root	[35]
**38**	Kirisine B	*Aconitum *kirinense** Nakai	root	[35]
**39**	Kirisine C	*Aconitum *kirinense** Nakai	root	[35]
**40**	Kirisine A	*Aconitum *kirinense** Nakai	root	[35]
**41**	Barpubenine A	*Aconitum *barbatum** var. *puberulum* Ledeb.	whole plant	[37]
**42**	Barpubenine B	*Aconitum *barbatum** var. *puberulum* Ledeb.	whole plant	[37]
**43**	1-N-deethyl-1,16-demethoxy-1,16-dihydroxyranaconidine	*Aconitum iochanicumone*	root	[38]

#### 2.3.2. C19-DAs (**44**–**182**)

C19-DAs are the largest class of DAs. According to the differences in the skeleton, they can be divided into six types: aconitines, lycoctonines, lactones, 7,17-seco, franchetines, and rearranged class [39]. Aconitines and lycoctonines constitute the majority of C19-DAs. This subsection summarizes 139 new C19-DAs discovered in the past five years, including 70 aconitine-type C19-DAs, 40 lycotonine-type C19-DAs, 5 lactone-type C19-DAs, 10 franchetine-type C19-DAs, 7 Seco-type C19-DAs, and 7 rearranged C19-DAs.

- Aconitine-type C19-DAs (**44**–**113**)

**Figure 4:**
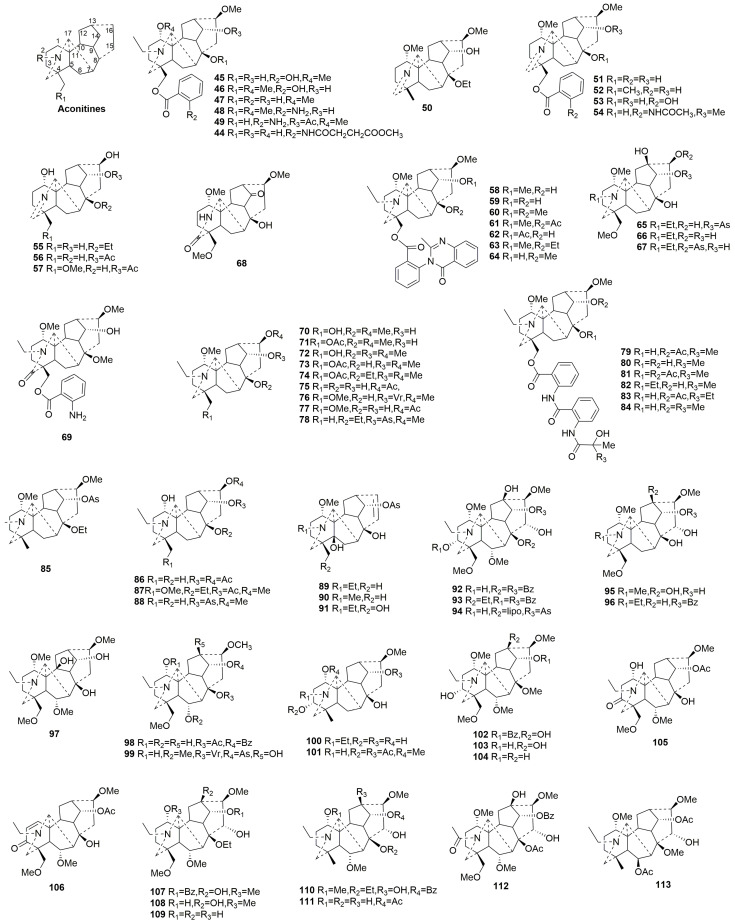
Structures of aconitine-type C19-DAs (**44**–**113**).

Aconitine-type C19-DAs are alkaloids characterized by the absence of an oxygen group at C-7. When ester groups are present at C-8 and C-14, they exhibit acute toxicity [8]. In the past five years, 70 new compounds (**44**–**113**) were discovered, with 65 obtained from *Aconitum* and 5 from *Delphinium* [32,34,38,40,41,42,43,44,45,46,47,48,49,50,51,52,53,54]. The compounds were further discussed according to the presence of oxygen-containing groups connected at C-3, C-6, or C-15 positions. The plant sources are shown in Table 4. Detailed chemical structures are shown in Figure 4.

Compounds **44**–**91** are alkaloids that lack oxygen-containing groups at C-3, C-6, and C-15. Notably, compounds **50**–**57**, featuring a double bond between the N and C-19, were isolated from *Aconitum* [38,40,50]. Compounds **58**–**64** are linked to a 2-(2-methyl-4-oxoquinazolin-3-yl) benzoate moiety at C-18 [49,54]. Compounds **58**–**63** exhibit rotational isomerism due to an unusual axial chiral phenyl-quinoline side chain at C-18, with the stereoisomerism studied by temperature-varying NMR techniques [49,54]. Compounds **68** and **69** have an unusual ketone group attached to C-19, with compound **68** uniquely featuring a ketone group at C-14 instead of a common methoxy or ester group. Compounds **79**–**84** are new C19-DAs with a vaginatunine A fragment at C-18 [32,55]. Compounds **89**–**91** lack a common methoxy group in DAs at C-16; however, there is a double bond between C-15 and C-16 [45,46,51].

Compounds **92**–**94** possess oxygen-containing groups at C-3, C-6, and C-15 [48,56,57]. Interestingly, compound **94** is the third reported C19-DA with hydroxyl groups at C-3, C-13, and C-15 and with a fatty acid ester moiety at C-8 [57]. Compounds **95**–**96** have hydroxyl groups at C-15 without oxygen-containing groups at C-3/C-6. Compounds **97**–**99** are DAs with oxygen-containing groups at C-6, while there are no oxygen-containing groups at C-3 and C-15. Compounds **100**–**101** have oxygen groups at C-3 but no oxygen groups at C-6 and C-15. Compounds **102**–**106** have oxygen-containing groups at C-3 and C-6, whereas **105** and **106** contain a ketone group at C-3 [42,48,58]. Compounds **107**–**113** have oxygen-containing groups at C-6 and C-15 but no oxygen-containing group at C-3 [59]. Interestingly, the acetyloxy group of compound **113** at the C-6 position is *β*-oriented, uncommon in aconitine-type DAs [48,59].

**Table 4:** Names and plant sources of aconitine-type C19-DAs (**44**–**113**).

No.	Compound Name	Sources	Plant Parts	Ref.
**44**	Naviconitine	*Delphinium naviculare* var. *lasiocarpum*	whole plant	[34]
**45**	Acoapetaludine D	*Aconitum apetalum* (Huth) B.Fedtsch	whole plant	[40]
**46**	Acoapetaludine E	*Aconitum apetalum* (Huth) B.Fedtsch	whole plant	[40]
**47**	Acoapetaludine F	*Aconitum apetalum* (Huth) B.Fedtsch	whole plant	[40]
**48**	Acoapetaludine G	*Aconitum apetalum* (Huth) B.Fedtsch	whole plant	[40]
**49**	Forrestline D	*Delphinium forrestii* var. *viride*	whole herb	[53]
**50**	Episcopaline C	*Aconitum episcopale*	root	[50]
**51**	Acoapetaludine H	*Aconitum apetalum* (Huth) B.Fedtsch	whole plant	[40]
**52**	Acoapetaludine I	*Aconitum apetalum* (Huth) B.Fedtsch	whole plant	[40]
**53**	Acoapetaludine J	*Aconitum apetalum* (Huth) B.Fedtsch	whole plant	[40]
**54**	Novolunine C	*Aconitum novoluridum*	root	[49]
**55**	Austroyunnanine C	*Aconitum austroyunnanense*	root	[45]
**56**	1-N-deethyl-1,16-demethoxy-1,16 dihydroxy-N(19)-en-austroconitine A	*Aconitum iochanicumone*	root	[38]
**57**	1-N-deethyl-1,16-demethoxy-1,16-dihydroxy-18-methoxy-N(19)-en-austroconitine A	*Aconitum iochanicumone*	root	[38]
**58**	Brevicanine A	*Aconitum *brevicalcaratum**	root	[54]
**59**	Novolunine A	*Aconitum novoluridum*	root	[49]
**60**	Novolunine B	*Aconitum novoluridum*	root	[49]
**61**	Brevicanine B	*Aconitum *brevicalcaratum**	root	[54]
**62**	Brevicanine C	*Aconitum *brevicalcaratum**	root	[54]
**63**	Brevicanine D	*Aconitum *brevicalcaratum**	root	[54]
**64**	Forrestline B	*Delphinium forrestii* var. *viride*	whole herb	[53]
**65**	Refractine A	*Aconitum refractum* var. circinatum	whole plant	[41]
**66**	Richardsonine B	*Aconitum richardsonianum* Lauener	root	[44]
**67**	Richardsonine C	*Aconitum richardsonianum* Lauener	root	[44]
**68**	Richardsonine A	*Aconitum richardsonianum* Lauener	root	[44]
**69**	Acoapetaludine K	*Aconitum apetalum* (Huth) B.Fedtsch	whole plant	[40]
**70**	Acoapetaludine B	*Aconitum apetalum* (Huth) B.Fedtsch	whole plant	[40]
**71**	Acoapetaludine C	*Aconitum apetalum* (Huth) B.Fedtsch	whole plant	[40]
**72**	Forrestline E	*Delphinium forrestii* var. *viride*	whole herb	[53]
**73**	Brevicalcarine B	*Aconitum brevicalcaratum*	root	[60]
**74**	Brevicalcarine C	*Aconitum brevicalcaratum*	root	[60]
**75**	Rockidine A	*Aconitum genera*	root	[43]
**76**	Pseudostapine A	*Aconitum pseudostapfianum*	root	[51]
**77**	Refractine B	*Aconitum refractum* var. circinatum	whole plant	[41]
**78**	Austroyunnanine A	*Aconitum austroyunnanense*	root	[45]
**79**	Apetalrine A	*Aconitum apetalum*	aerial part	[55]
**80**	Apetalrine B	*Aconitum apetalum*	aerial part	[55]
**81**	Apetalrine C	*Aconitum apetalum*	aerial part	[55]
**82**	Apetalrine D	*Aconitum apetalum*	aerial part	[55]
**83**	Apetalrine E	*Aconitum apetalum*	aerial part	[55]
**84**	Brevicalcarine A	*Aconitum brevicalcaratum*	root	[60]
**85**	Nagarumine A	*Aconitum nagarum*	root	[46]
**86**	Nagarutine A	*Aconitum nagarum* Stapf	root	[42]
**87**	Episcopaline A	*Aconitum episcopale*	root	[50]
**88**	Pseudostapine B	*Aconitum pseudostapfianum*	root	[51]
**89**	Pseudostapine C	*Aconitum pseudostapfianum*	root	[51]
**90**	Nagarumine B	*Aconitum nagarum*	root	[46]
**91**	Austroyunnanine B	*Aconitum austroyunnanense*	root	[45]
**92**	Smirnotine A	*Aconitum smirnovii* Steinb	aerial part	[58]
**93**	Pendulumine A	*Aconitum* pendulum	rhizome	[48]
**94**	Lipojesaconitine	*Aconitum japonicum* subsp. *subcuneatum*	rhizoma	[57]
**95**	6-demethoxyhypaconine	*Aconitum carmichaelii* Debx.	lateral root	[47]
**96**	Carmichaeline K	*Aconitum carmichaelii* Debx.	lateral root	[47]
**97**	10-hydroxychasmanine	*Aconitum japonicum* subsp. *subcuneatum*	rhizoma	[57]
**98**	Rockidine B	*Aconitum genera*	root	[43]
**99**	Geordine	*Aconitum georgei* Comber	root	[61]
**100**	3-hydroxykaracoline	*Aconitum japonicum* subsp. *subcuneatum*	rhizoma	[57]
**101**	Episcopine B	*Aconitum episcopale*	root	[52]
**102**	Acotarine F	*Aconitum* taronense	root	[56]
**103**	Acotarine G	*Aconitum* taronense	root	[56]
**104**	Smirnotine B	*Aconitum *smirnovii** Steinb	aerial part	[58]
**105**	Pendulumine E	*Aconitum *pendulum**	rhizome	[48]
**106**	Nagarutine C	*Aconitum nagarum* Stapf	root	[42]
**107**	8-*O*-ethyl-benzoyldeoxyaconine	*Aconitum carmichaelii* Debx.	lateral root	[47]
**108**	Pendulumine C	*Aconitum* pendulum	rhizome	[48]
**109**	Pendulumine D	*Aconitum* pendulum	rhizome	[48]
**110**	Nagarutine B	*Aconitum nagarum* Stapf	root	[42]
**111**	Nagarutine D	*Aconitum nagarum* Stapf	root	[42]
**112**	Pendulumine F	*Aconitum* pendulum	rhizome	[48]
**113**	Delcarpum	*Delphinium peregrinum* L. var. *eriocarpum Boiss*	aerial part	[59]

- Lycoctonine-type C19-DAs (**114**–**153**)

**Figure 5:**
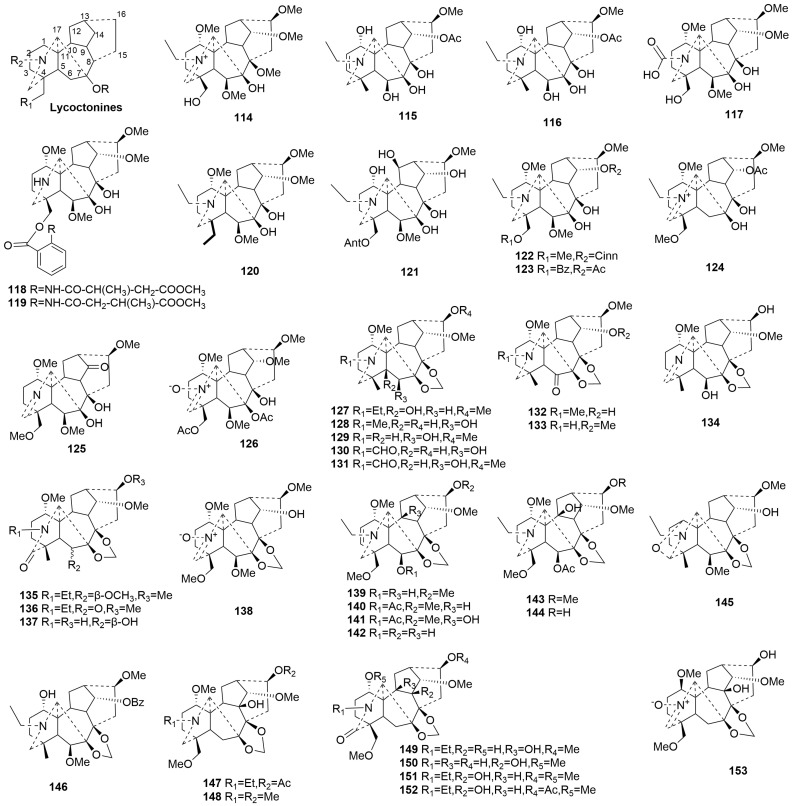
Structures of lycoctonine-type C19-DAs (**114**–**153**).

Lycoctonine-type C19-DAs are oxidized at C-7 and C-8. Compounds **114**–**153** are all novel members of the lycoctonine-type C19-DAs, with 38 compounds derived from *Delphinium* and only 2 from *Aconitum* [62,63,64,65,66,67,68]. The plant sources are shown in Table 5. Detailed chemical structures are shown in Figure 5.

According to the different oxygen-containing groups at C-7 and C-8, they can be divided into two subtypes. Eleven new compounds (**115**–**125**) feature a C-7 and C-8 diol. Compounds **114** and **126** have rare methoxy and acetoxy groups at C-8, respectively [34,69]. Compounds **115** and **116,** isolated from *Aconitum *sczukinii,** have very similar chemical structures, with the only difference being the presence of double bonds between C-2 and C-3 in compound **115** [65]. Compound **117** contains a carboxyl group attached to the nitrogen. Compounds **118** and **119** are identified as a pair of regioisomers [62]. Compound **121** from *Delphinium ajacis* is notable for its rare hydroxyl group at C-12 [70]. Compounds **124**–**126** all featured a characteristic N=CH fragment, with compound **126** also possessing an additional nitrone group [68].

Twenty-seven novel compounds (**127**–**153**) obtained from *Delphinium* species all have a 7,8-methylenedioxy group. Compounds **130** and **131** each have a rare aldehyde group attached to the N atom [67]. Compound **145** is unprecedented, with an ether bond between C-1 and C-19 [71]. Compounds **134**, **138**, and **153** have an N=CH fragment, with compounds **138** and **153** further possessing a nitrone group [64,67,72]. Compounds **136**–**137** and **149**–**152** have a characteristic keto group attached to C-19 [67,72]. Interestingly, compound **153** from *Delphinium* displays an unusual *β*-oriented 1-OMe [72].

**Table 5:** Names and plant sources of lycoctonine-type C19-DAs (**114**–**153**).

No.	Compound Name	Sources	Plant Parts	Ref.
**114**	Naviculine	*Delphinium naviculare* var. *lasiocarpum*	whole plant	[34]
**115**	Sczukiniline D	*Aconitum sczukinii* Turcz	root	[65]
**116**	Sczukiniline E	*Aconitum sczukinii* Turcz	root	[65]
**117**	Grandifline C	*Delphinium grandiflorum*	aerial parts	[73]
**118**	Shawurenine C	*Delphinium shawurense* W. T. Wang	aerial parts	[62]
**119**	Shawurenine D	*Delphinium shawurense* W. T. Wang	aerial parts	[62]
**120**	Uncinatine-A	*Delphinium uncinatum*	whole plant	[66]
**121**	Ajacisine G	*Delphinium ajacis*	seed	[70]
**122**	Grandiflonine F	*Delphinium* grandiflorum L.	whole plant	[68]
**123**	Ajacisine F	*Delphinium ajacis*	seed	[70]
**124**	Grandiflonine E	*Delphinium* grandiflorum L.	whole plant	[68]
**125**	Grandiflonine G	*Delphinium* grandiflorum L.	whole plant	[68]
**126**	Chrysotrichumine A	*Delphinium chrysotrichum*	aerial parts	[69]
**127**	Elapaciline	*Delphinium elatum* cv. Pacific Giant	seed	[67]
**128**	Meladine	*Delphinium elatum* cv. Pacific Giant	seed	[67]
**129**	N-deethyldelpheline	*Delphinium elatum* cv. Pacific Giant	seed	[67]
**130**	N-deethyl-N-formyleladine	*Delphinium elatum* cv. Pacific Giant	seed	[67]
**131**	N-deethyl-N-formyldelpheline	*Delphinium elatum* cv. Pacific Giant	seed	[67]
**132**	Melapacitine	*Delphinium elatum* cv. Pacific Giant	seed	[67]
**133**	N-deethylpacinine	*Delphinium elatum* cv. Pacific Giant	seed	[67]
**134**	Iminoeladine	*Delphinium elatum* cv. Pacific Giant	seed	[67]
**135**	19-oxopaciline	*Delphinium elatum* cv. Pacific Giant	seed	[67]
**136**	19-oxopacinine	*Delphinium elatum* cv. Pacific Giant	seed	[67]
**137**	N-deethyl-19-oxoeladine	*Delphinium elatum* cv. Pacific Giant	seed	[67]
**138**	Brunodelphinine C	Delphinium brunonianum Royle	aerial parts	[64]
**139**	Grandifloline A	*Delphinium grandiflorum* L.	whole herb	[63]
**140**	Grandifloline B	*Delphinium grandiflorum* L.	whole herb	[63]
**141**	Grandifloline C	*Delphinium grandiflorum* L.	whole herb	[63]
**142**	Grandifloline E	*Delphinium grandiflorum* L.	whole herb	[63]
**143**	Grandifloline D	*Delphinium grandiflorum* L.	whole herb	[63]
**144**	Grandifloline F	*Delphinium grandiflorum* L.	whole herb	[63]
**145**	Liangshanine A	*Delphinium liangshanense* W. T. Wang	whole plant	[71]
**146**	Liangshanine B	*Delphinium liangshanense* W. T. Wang	whole plant	[71]
**147**	Kamaonensine A	*Delphinium kamaonense* Huth	whole plant	[72]
**148**	Kamaonensine B	*Delphinium kamaonense* Huth	whole plant	[72]
**149**	Kamaonensine C	*Delphinium kamaonense* Huth	whole plant	[72]
**150**	Kamaonensine D	*Delphinium kamaonense* Huth	whole plant	[72]
**151**	Kamaonensine E	*Delphinium kamaonense* Huth	whole plant	[72]
**152**	Kamaonensine G	*Delphinium kamaonense* Huth	whole plant	[72]
**153**	Kamaonensine F	*Delphinium kamaonense* Huth	whole plant	[72]

- Lactone-type C19-DAs (**154**–**158**)

**Figure 6:**
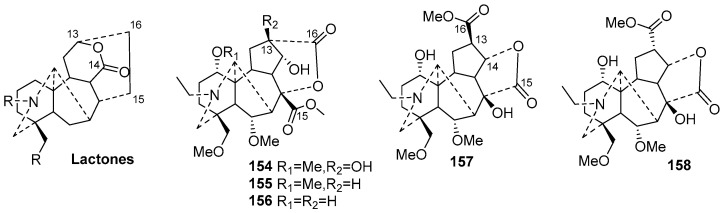
Structures of lactone-type C19-DAs (**154**–**158**).

Lactone-type C19-DAs are generally formed by oxidation of the 14-ketone in the C ring of aconitine-type DAs to form a six-membered lactone C ring. Only five new members (**154**–**158**) belong to this type. The plant sources are shown in Table 6. Detailed chemical structures are shown in Figure 6.

Interestingly, these five newly discovered lactone-type C19-DAs have an unprecedented five-membered lactone D ring [74,75]. Compounds **154**–**158** are formed by cleavage of the bond between C-15 and C-16, followed by subsequent lactonization. In addition, compounds **157** and **158** are C-13 epimers of each other, highlighting a unique aspect of their structural configuration [74].

**Table 6:** Names and plant sources of lactone-type C19-DAs (**154**–**158**).

No.	Compound Name	Sources	Plant Parts	Ref.
**154**	Kusnezosine A	*Aconitum kusnezoffii* Reichb. var. *gibbiferum*	root	[75]
**155**	Kusnezosine B	*Aconitum kusnezoffii* Reichb. var. *gibbiferum*	root	[75]
**156**	Kusnezosine C	*Aconitum kusnezoffii* Reichb. var. *gibbiferum*	root	[75]
**157**	Stylosine A	*Aconitum stylosum*	root	[74]
**158**	Stylosine B	*Aconitum stylosum*	root	[74]

- Franchetine-type C19-DAs (**159**–**168**)

**Figure 7:**
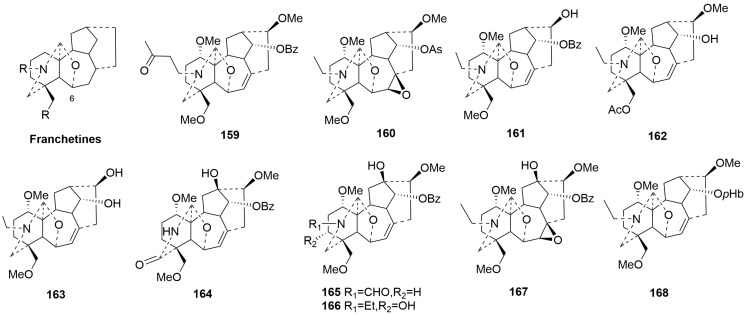
Structures of franchetine-type C19-DAs (**159**–**168**).

Franchetine-type C19-DAs are distinguished from aconitines by an additional ether between C-6 and C-17. Ten novel alkaloids (**159**–**168**) are classified as franchetine-type C19-DAs [43,56,76]. The plant sources are shown in Table 7. Detailed chemical structures are shown in Figure 7. Compounds **159**, **161**–**166**, and **168** have a double bond between C-7 and C-8, whereas compounds **160** and **167** have a 7,8-epoxy unit [43,56,76]. In addition, Compounds **161** and **163** have a characteristic hydroxyl group at C-16 instead of the methoxy group joint in DAs.

- 7,17-*seco*-type C19-DAs (**169**–**175**)

**Figure 8:**
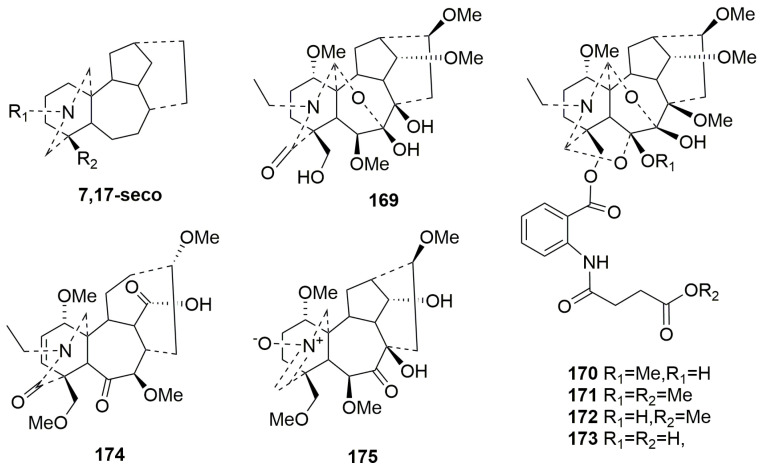
Structures of 7,17-*seco*-type C19-DAs (**169**–**175**).

7,17-*seco*-type C19-DAs are characterized by the cleavage of the C7-C17 bond, typically along with double bonds between C-7 and C-8. There are seven members (**169**–**175**) of this class that have been identified over the past five years. Interestingly, compounds **169**–**173** have a hemiacetal fragment, which results from the C7-C17 bond breaking, followed by the formation of an ether bond [64,73,77]. Compounds **169**–**173** are the 7,17-secolycoctonine C19-DAs with a C7-O-C17 unit [73]. Compounds **170**–**173** have an unprecedented N, O-diacetyl residue [77]. Among them, compounds **170** and **172** are isomers; the methoxy group is located at C-11 in **172** and at C-6 in **170**. Moreover, compound **173**, lacking a methoxy group at C-6, is a demethylation product of **170**. The plant sources are shown in Table 8, and detailed chemical structures are shown in Figure 8.

- Rearranged C19-DAs (**176**–**182**)

**Figure 9:**
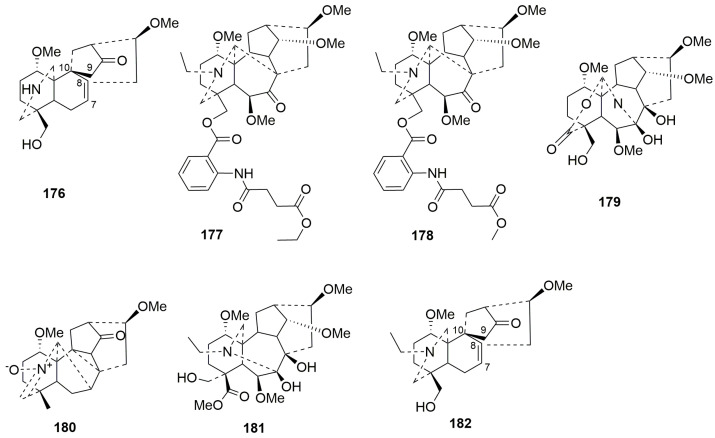
Structures of rearranged C19-DAs (**176**–**182**).

Over the past five years, only seven members (**176**–**182**) have been classified as rearranged C19-DAs [46,52,64,73,78]. Compounds **176** and **182** are unique alkaloids with a rearranged six-membered B ring formed by the C-8 and C-10 linkage [46,50]. Compounds **177** and **178** are two new rare rearranged aconitine-type C19-diterpenoid alkaloids whose C7-C17 bond rearranges to form a C8-C17 bond [79]. The N-C19 and C7-C17 bonds in compound **179** are broken and rearranged into N-C7 bonds, and C-19 was oxidized to carbonyl [73]. In addition, compound **179** belongs to a new rearranged subtype named grandiflodines, which possesses a C7-N-C17 unit and a C17-O-C19 unit. Compound **181** displays an unusual rearranged C19-DA skeleton with the cleavage of N-C19 and C7-C17 bonds and the construction of the N-C7 bond [78]. The plant sources are shown in Table 9. Detailed chemical structures are shown in Figure 9.

#### 2.3.3. C20-DAs (**183**–**266**)

C20-DAs are compounds with structures more complex than those of C18- and C19-DAs, with most C20-DAs having a characteristic exocyclic double bond between C-16 and C-17. Based on the variations in the skeletons, the vast majority of C20-DAs can be classified into seven types: atisines, deudatines, hetisines, hetidines, anopterines, napellines, and vakognavines [39]. This section describes 84 C20-DAs from nature, including 9 atisine-type C20-DAs, 26 hetisine-type C20-DAs, 11 hetidine-type C20-DAs, 13 deudatine-type C20-DAs, 8 napelline-type C20-DAs, 6 vakognavine-type C20-DAs, and 11 rearranged C20-DAs. Among these alkaloids, 62 were obtained from *Aconitum* plants and 22 from *Delphinium* plants.

- Atisine-type C20-DA (**183**–**191**)

**Figure 10:**
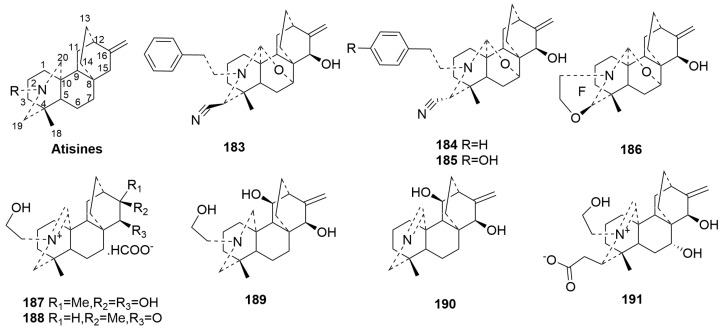
Structures of atisine-type C20-DAs (**183**–**191**).

Atisine-type C20-DAs are structurally characterized by their N atoms being linked to C-20 and C-19 and share the same carbon skeleton as atisine diterpenes. Only nine newly discovered compounds (**183**–**191**) have been classified as atisine-type C20-DAs [37,53,69,80,81]. Compounds **183**–**186**, derived from *Delphinium*, feature an additional ether bond between C-20 and C-7. Among these, compounds **183**–**185** have cyano groups at C-19, making the first reported cyano-containing DAs [80]. Furthermore, compound **186** is noted for bearing an oxazolidine ring F [69]. Compounds **187**–**188** and **190**–**191** have a double bond between N and C-20, with compound **188** possessing a rare ketone group at C-15 [37]. The plant sources are shown in Table 10. Detailed chemical structures are shown in Figure 10.

- Hetisine-type C20-DAs (**192**–**217**)

**Figure 11:**
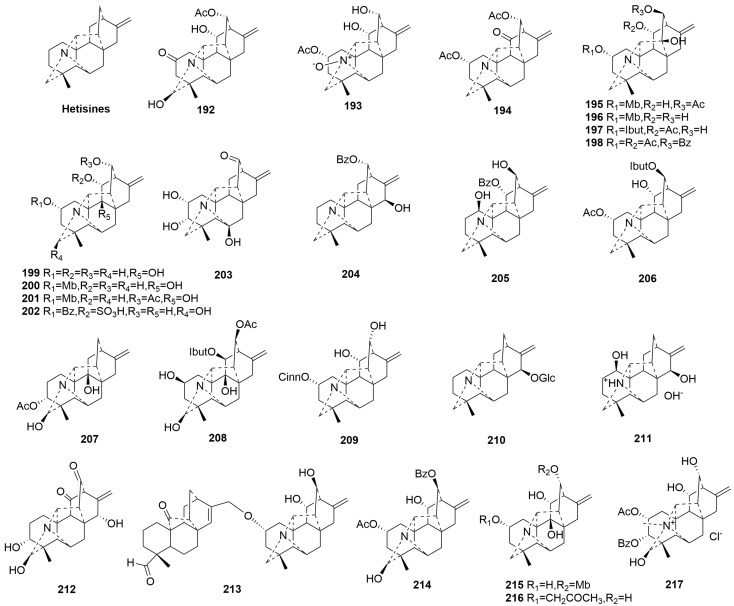
Structures of hetisine-type C20-DAs (**192**–**217**).

Compared to atisine-type C20-DAs, hetisine-type C20-DAs feature a hexacyclic with an additional C14-C20 bond and N-C6 bond [82]. This category is the largest, and the newly discovered compounds **192**–**217** belong to this class of C20-DAs [37,59,68,83,84]. Among these, only compound **203**, obtained from *Aconitum*, has a hydroxyl group at C-6 [85]. Compounds **204**–**206** and **211**–**212** have a hydroxyl group at C-15; notably, only compound **212** shows an *α*-oriented OH group at C-15 [86]. Compound **210** is the first hetisine-type C20-DA with one hexose substitution, identified as *β*-glucoside [47]. In addition, Compound **213** is a rare DA linked by the ether bond between C-17′ and C-2 between hetisine-type C20-DAs and hetidane-type diterpenes [84]. The plant sources are shown in Table 11. Detailed chemical structures are shown in Figure 11.

- Hetidine-type C20-DAs (**218**–**228**)

**Figure 12:**
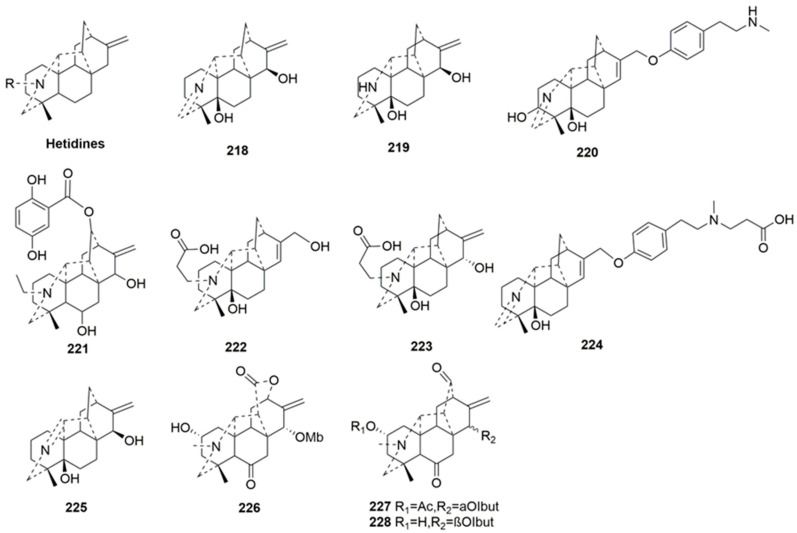
Structures of hetidine-type C20-DAs (**218**–**228**).

Hetidine-type C20-DAs possess an additional C14-C20 bond, distinguishing them structurally from the atisine class. Eleven new members (**218**–**228**) have been classified as such [65,81,84,85,90,91]. Compounds **218**–**219**, **221**, **223**, and **225**–**226** all feature a typical exocyclic double bond characteristic of C20-DAs, while compounds **220**, **222**, and **224** exhibit a distinct intra-ring double bond between C15 and C16. Moreover, compound **226** is notable for having an ester group between C-12 and C-14, forming a lactone ring D, representing a novel skeleton of hetidine-type C20-DAs [65]. The plant sources are shown in Table 12. Detailed chemical structures are shown in Figure 12.

- Denudatine-type C20-DAs (**229**–**241**)

**Figure 13:**
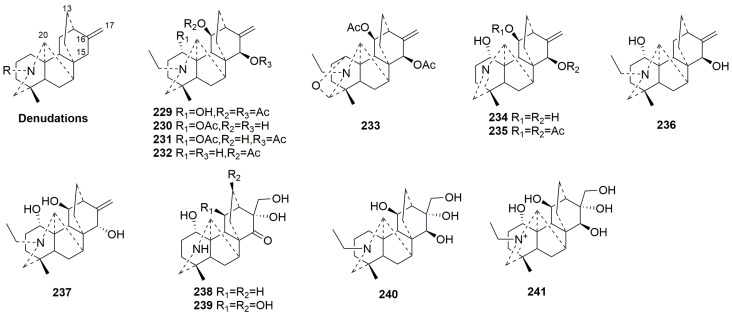
Structures of denudatine-type C20-DAs (**229**–**241**).

Denudatine-type C20-DAs possess an additional C7-C20 bond compared to the atisine class. Thirteen new compounds (**229**–**241**) isolated from the *Aconitum* genus have been classified as this class [35,37,93]. Compounds **229**–**237** possess typical exocyclic double bonds between C-16 and C-17, while compounds **238**–**241** display a hydroxyl group at C-16 and C-17. Compound **233** includes a rare ether bond between C-1 and C-19 [35]. The plant sources are shown in Table 13. Detailed chemical structures are shown in Figure 13.

- Napelline-type C20-DAs (**242**–**249**)

**Figure 14:**
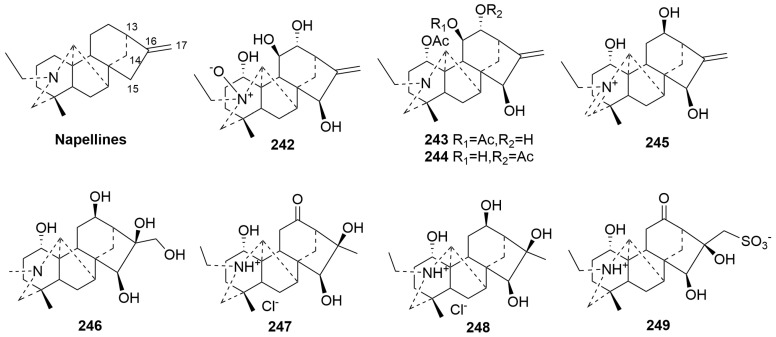
Structures of napelline-type C20-DAs (**242**–**249**).

Napelline-type C20-DAs are structurally similar to kaurane diterpenes with the distinctive additional C7-C20 bond. The new compounds **242**–**249** obtained from the genus *Aconitum* have been classified as this class [35,94,95,96]. Compounds **242**–**245** have a typical exocyclic double bond. Among these, compound **242** is a rare N-oxide of natural napelline-type C20-DAs, and compound **245** presents a C20-DA with an iminium methine moiety [35,96]. Compounds **247**–**248** are napelline-type hydrochloride C20-DAs with a characteristic methyl and hydroxyl group at C-16 instead of a typical exocyclic double bond [94]. Compound **249** contains a sulfonic acid unit [95]. The plant sources are shown in Table 14. Detailed chemical structures are shown in Figure 14.

- Vakognavine-type C20-DAs (**250**–**255**)

**Figure 15:**
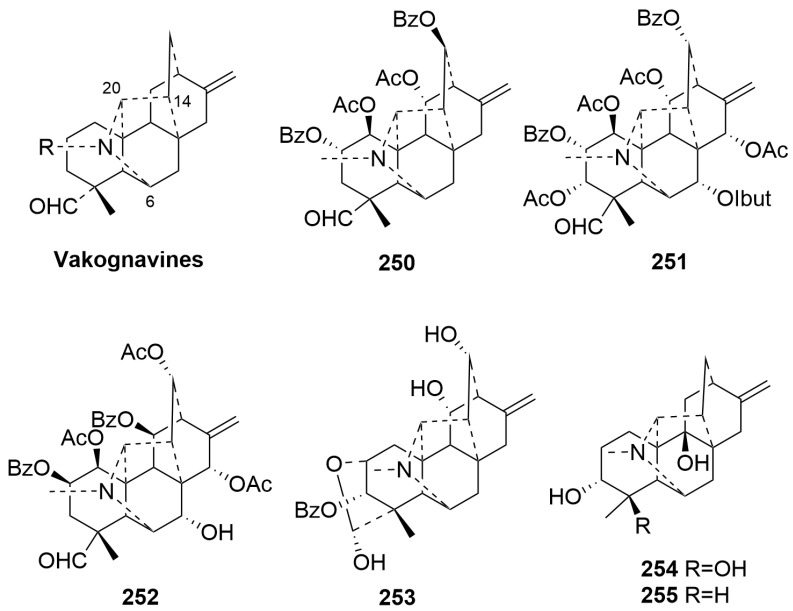
Structures of vakognavine-type C20-DAs (**250**–**255**).

The fundamental skeleton structure of vakognavine-type C20-DAs is defined by the bond cleavage between N and C-19 of hetisines. Only six new compounds (**250**–**255**) belong to this class [43,68,89,97]. Compound **253** is the first vakognavine-type C20-DA with a characteristic C2-O-C19 unit. Compounds **254** and **255** represent the first natural diterpenoid alkaloid at C-18, with an α-oriented methyl group [68]. The plant sources are shown in Table 15. Detailed chemical structures are shown in Figure 15.

- Rearranged C20-DAs (**256**–**266**)

**Figure 16:**
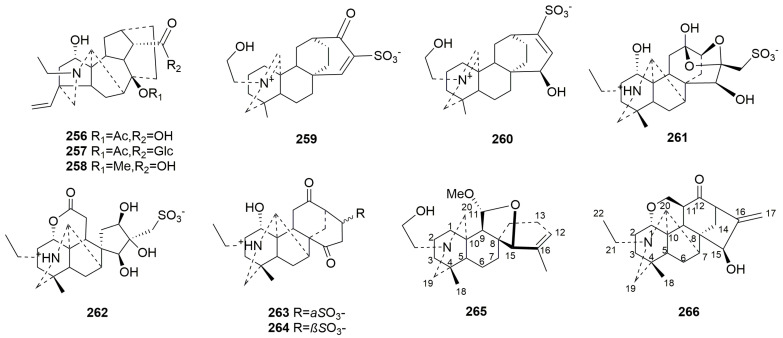
Structures of rearranged C20-DAs (**256**–**266**).

Rearranged C20-DAs retain the characteristics of C20-DAs but with altered skeletons. Eleven new compounds (**256**–**266**) obtained from *Aconitum* belong to this class [37,40,84,95,98,99]. Compounds **256**–**258** are rearranged C20-DAs with racemulosine skeletons derived from denudatine–type DAs via double Wanger–Meerwein rearrangements of rings A and C [84]. Compounds **259**–**260** are zwitterionic sulfonated C20-DAs with a rearranged atisane skeleton. Compounds **261**–**262** are two sulfonated seco C20-DAs originating from the Criegee rearrangements; notably, compound **261** is a 13,16-*seco*-napelline DA, and **262** is a 12,13-*seco*-napelline DA [99]. Compounds **263**–**264** are novel DAs derived through semipinacol rearrangements of the napelline-type DAs, which migrate through C13−C16 and C15−C6 bonds, respectively [95]. Compound **266** has a tetra-hydropyran ring system unlike napellines [40]. The plant sources are shown in Table 16. Detailed chemical structures are shown in Figure 16.

#### 2.3.4. Bis-DAs (**267**–**280**)

**Figure 17:**
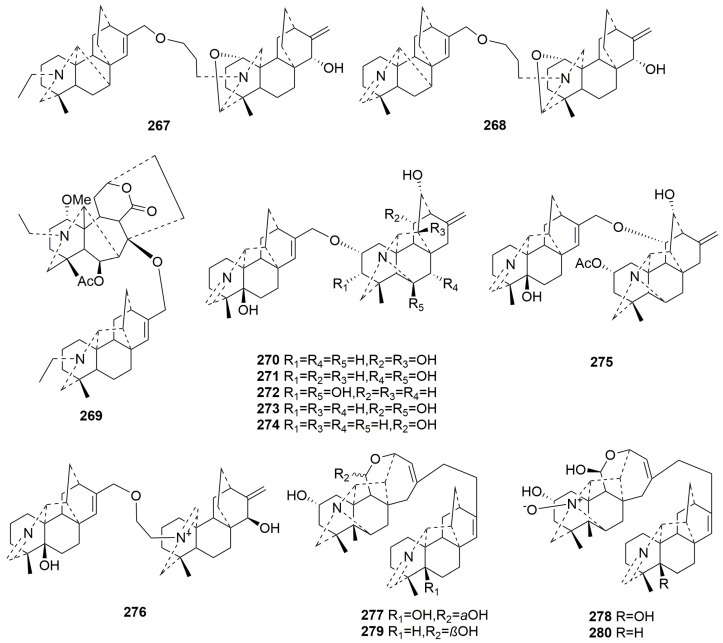
Structures of Bis-DAs (**267**–**280**).

Bis-DAs are formed by condensing two molecules of diterpenoid alkaloids, typically linked via an O-ether linkage. Fourteen new members (**267**–**280**), all obtained from the *Aconitum* plant, have been identified [84,92,100]. The plant sources are shown in Table 17. Detailed chemical structures are shown in Figure 17.

Compounds **267**–**268** are classified as denudatine–atistine-type Bis-DAs, with denudatine and atistine fragments linked by an ether bond between C-17 and C-22′ [100]. Compound **269**, with heteratisine and hetidine fragments linked by an ether bond between C-17 and C-18′, falls into the heteratisine–hetidine class of alkaloids [92]. Compounds **270**–**275** belong to hetidine–hetisine class, whose hetidine fragments and hetisine fragments are linked by ether bonds [84]. Among these, Compounds **270**–**274** are isomers, differing only in the position of the hydroxyl substitution in the hetisine moiety [92]. Compounds **277**–**280**, as hetidine-rearranged hetisine-class alkaloids, are bridged by a rare single bond between C-17 and C-17′ in Bis-DAs [84,92].

#### 2.3.5. Other DAs (**281**–**289**)

**Figure 18:**
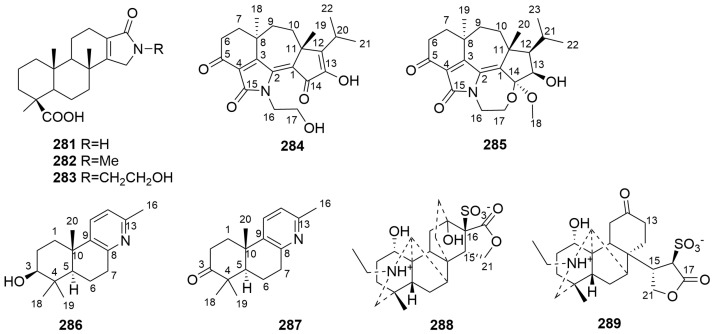
Structures of other DAs (**281**–**289**).

In addition to the diterpenoid alkaloids mentioned above, some diterpenoid alkaloids have novel structures, such as compounds **281**–**289** [100,101,102,103]. The sources are shown in Table 18. Detailed chemical structures are shown in Figure 18.

Compounds **281**–**283**, isolated from the marine sponge *Spongia* sp., a marine invertebrate, represent three novel DAs with *γ*-lactam rings [101]. Compounds **284**–**285** are novel DAs with a guanacastane skeleton isolated from the endophytic fungus *Trichoderma koningii* A729 [102].

Compounds **286**–**287** are two C17-labdane DAs isolated from ripe fruits of *Forsythia suspensa*. The structure of **286** was determined as 3-hydroxyl-4,4,10,13-tetramethyl-1(2),3(4),5(10),6(7)-octahydrobenzo[f]quinolin, and **287** was obtained by oxidation of the hydroxyl group at C-3 in **286** [103]. Compounds **288**–**289**, isolated from lateral roots of *Aconitum carmichaelii*, represent the first reported natural C21-DAs. The structure of **288** is similar to that of denudatine- and napelline-type C20-DAs. Compound **289** is an isomer of **288**, a 12,16-*seco* derivative of **288** [104].

## 3. Biological Activity

Terpenoid alkaloids, a class of compounds with far-reaching pharmacological significance, exhibit unique pharmacological effects and extensive biological activities. This section provides an overview of the biological activities of TeAs that have been newly discovered in the past five years, including anti-inflammatory activity, analgesic effect, anticancer activity, and antibacterial and antiviral properties. A table of TeAs’ biological activities is provided (Table 19).

### 3.1. Anti-Inflammatory Activity

The anti-inflammatory activity of TeAs has been well documented, with compounds like gentianine and benzoylaconitine among those reported [105,106]. This paper highlights 16 new anti-inflammatory members in TeAs over the past five years, including 4 monoterpene alkaloids and 12 DAs.

Delavatines C (**9**) and E (**11**) showed more significant inhibition of NO production in lipopolysaccharide (LPS)-stimulated BV2 cells compared to aminoguanidine bicarbonate, with IC_50_ values of 25.62 and 17.29 μM, respectively, and no cytotoxicity [18]. Stylosine A (**157**) showed significant inhibitory activity against LPS-induced production of inflammatory cytokines (IL-1*β*, COX-2, and TNF-*α*) in RAW264.7 cells at a dose of 0.1 μg/mL without cytotoxicity [74]. Geordine (**103**) exhibited specific anti-inflammatory activity and inhibited LPS-induced NO production in RAW264.7 cells at 50 μM, with an inhibition rate of 29.75% [61]. Ajacisines F-H (**121**, **123,** and **214**) showed strong anti-inflammatory activity by inhibiting LPS-induced NO production in BV-2 cells, with inhibition rates of 80% at 50 μM and no cytotoxicity [70]. Anthoroidine B (**277**) inhibited the production of NO and TNF-*α*, with IC_50_ values of 357.68 and 67.56 μM, respectively [84].

Forqinlingines C-D (**14**–**15**) and forsyqinlingines A-B (**286**–**287**) showed anti-inflammatory activities by inhibiting the release of *β*-glucuronidase in polymorphonuclear leukocytes (PMNs) induced by platelet-activating factor (PAF), with inhibition rates of 45.2%, 40.1%, 56.7%, and 58.6%, respectively [21].

Kamaonensines B (**148**) and F (**153**) showed more robust anti-inflammatory activities than the positive drug indomethacin (9.0 ± 1.3 μM), with IC_50_ values of 2.7 ± 0.5 and 0.9 ± 0.2 μM, respectively. Network pharmacological studies indicated that the anti-inflammatory mechanism may be related to the MAPK signaling pathway. In addition, molecular docking results showed that the infrequent amides and methylenedioxy groups could be the two critical pharmacophores in **148** and **153** [72].

Aconicumine A (**170**) exhibited anti-inflammatory activity by inhibiting LPS-activated NO production in RAW264.7 cells (IC_50_ = 19.7 ± 1.1 μM). Structure–activity relationship studies identified the methoxy group at its C-6 position is an effective group for anti-inflammatory activity [77].

Forrestline F (**190**) significantly inhibited NO activity in RAW264.7 cells (IC_50_ = 9.57 ± 1.34 μM). Further studies showed modulating anti-inflammatory effects through inhibiting ROS production and NF-*κ*B, MAPK, and Nrf2 signaling pathways [53].

### 3.2. Analgesic Activity

Opioids and non-steroidal anti-inflammatory drugs (NSAIDs) are the primary drugs for pain treatment [107]. However, both drug classes can cause severe adverse reactions in clinical use. As an essential class of TeAs, the analgesic activity of DAs has been widely studied. Several new compounds with analgesic activity have been reported in the past five years of research. Compounds **89**, **91**, **176**, **180**, **182**, **241**, **259**–**264**, and **288** showed analgesic activity by inhibiting acetic acid-induced abdominal contractions in mice.

Compounds **89**, **91**, **176**, **180**, and **182** exhibited more excellent analgesic activity than the positive controls aspirin and acetaminophen. Episcopaline B (**182**), Pseudostapine C (**89**), Austroyunnanine B (**91**), and Episcopine A (**180**) significantly reduced acetic acid-induced abdominal contractions in mice in a dose-related manner, with the ID_50_ values of 55.0, 60.3, 48.0, and 66.1 μmol/kg, respectively [45,50,51,52]. Nagarumine C (**176**) demonstrated significant analgesic activity and inhibited acetic acid-induced writhing in mice at 76.0 μmol/kg [46].

Aconicatisulfonines A (**259**) and B (**260**) showed significant analgesic activity against acetic acid-induced writhing in mice, with inhibition rates of 43.2% and 64.7% (morphine, 66.8%) at 0.3 mg/kg, respectively [98]. Aconicarnine E (**241**) inhibited acetic acid-induced writhing in mice by 43.8% at 1.0 mg/kg [93]. Aconapelsulfonines A (**261**) and B (**262**) showed specific analgesic effects at the dose of 0.3 mg/kg, with inhibition rates of 63.6% and 19.3% (morphine, 84.6%), respectively [99]. Aconicarmisulfonines B (**263**) and C (**264**) displayed analgesic effects in mice, with inhibition rates of 31.26% and 26.84%, respectively [95]. Aconidenusulfonine A (**288**) showed analgesic activity, reducing acetic acid-induced writhing in mice by 26.35% at 2.0 mg/kg (i.p.), and its structure-activity relationship indicated that the analgesic activity might be related to a single bond between C-12 and C-16 [104].

The transient receptor vanilloid 1 (TRPV1) channel is a crucial target in developing new analgesics for pain management [108]. Acosinomonine B (**178**) showed a strong inhibitory effect on the activation of the TRPV1 channel in HEK-293 cells mediated by capsaicin (0.5 μM), with an inhibition rate of 31.78% at the concentration of 10 μM, making compound **178** a promising analgesic lead structure [79].

### 3.3. Antitumor Activity

TeAs have proven to be effective chemotherapeutic drugs for various cancers. For example, paclitaxel and its derivatives docetaxel and cabazitaxel have been clinically used for cancer treatment [109]. Over the past five years, studies have identified eight new members of TeAs with potential anticancer activity.

Incarvine G (**12**) showed cytotoxicity with the IC_50_ value of 60.29 μM against MDA-MB-231 cells and inhibited the migration and invasion of breast cancer cells. Further mechanistic studies showed that Incarvine G inhibited the migration and invasion of MDA-MB-231 cells by inhibiting actin cytoskeleton formation [19]. (±)-Caryopterisines A (**19**) and B (**20**) reduced kynurenine (Kyn) biosynthesis in HeLa cells by inhibiting indoleamine 2,3-dioxygenase (IDO) at doses of 10 μM with inhibition ratios of 25.7% and 29.8%, respectively [22]. Given the role of IDO cancer immunotherapy [110], compounds **19** and **20** are highlighted for their potent anticancer activities via IDO inhibition.

Commipholactam A (**27**) showed cytotoxicity against HepG2 and A549 cells, with IC_50_ values of 21.73 ± 2.86 μM and 128.50 ± 17.06 μM, respectively [28]. 8-O-ethyl-benzoyldeoxyaconine (**107**) demonstrated strong anticancer activity with an IC_50_ of 12.58 ± 1.82 and 12.76 ± 2.10 μM against human non-small-cell lung cancer A549 and H460 cells, respectively [47]. Lipojesaconitine (**98**) displayed significant cytotoxicity against four cell lines (A549, MDA-MB-231, MCF-7, and KB) with IC_50_ values ranging from 6.0 to 7.3 μM. However, it showed weak cytotoxicity against KB-VIN (IC_50_ = 18.6 μM), suggesting potential efflux by P-gp [57].

Brunonianines B (**182**) and C (**183**) showed significant cytotoxicity on Caco-2 (colon cancer) and Skov-3 (human ovarian cancer) cell lines. Compounds **182** and **183** showed comparable cytotoxicity to hydroxycamptothecin (HCPT) against the Caco-2 cell line, with IC_50_ values of 3.14 ± 0.37 and 2.41 ± 0.35 μM, respectively. Moreover, compound **182** showed stronger cytotoxicity than the HCPT (2.29 μM) Skov-3 cell line, with an IC_50_ value of 2.20 μM, likely due to its 19-S conformation. Further mechanistic studies showed that compound **182** could activate the Bax/Bcl-2/caspase-3 signaling pathway to induce apoptosis and effectively inhibit Skov-3 cell proliferation, migration, and invasion [80]. Ceylonamide G (**281**) was cytotoxic to DU145 cells, a human prostate cancer cell line, with an IC_50_ value of 6.9 μM and a minimum effective concentration (MEC) of 10 μM [101].

### 3.4. Cardioprotective Activity

Cardioprotective activity is a unique biological activity of DAs, such as Guan fu base A, which has been clinically developed to treat arrhythmias. Two new DAs with cardioprotective activity have been discovered in the past five years.

Smirnotine A (**94**) has some preventive effects on aconitine-induced arrhythmia in mice. The occurrence of ventricular tachycardia and ventricular flutter was significantly prolonged at 8 mg/kg, and ventricular flutter, ventricular fibrillation, and survival time of mice were prolonged considerably at 16 mg/kg [58]. Gyalanunine A (**253**) showed significant cardiotonic activity after perfusion in frog hearts and significantly inhibited myocardial contraction when combined with *β*-blockers in isolated frog hearts, suggesting that its mechanism of action may be related to epinephrine *β* receptors [89]. The existence of a hemiacetal moiety might be the critical structural feature necessary for the cardiac effect of **253** [89].

### 3.5. Antimicrobial Activity

Natural alkaloids have been proven to possess excellent antimicrobial activity. In the past five years, it has been found that several TeAs exhibit antimicrobial activity, such as antibacterial, antiviral, and antiplasmodial activities.

#### 3.5.1. Antiviral Activity

Forsyqinlingines C-D (**14**–**15**) and forsyqinlingine A-B (**286**–**287**) showed antiviral activities against influenza A virus (H1N1) and respiratory syncytial virus (RSV). They exhibited IC_50_ values of 11.9, 15.1, 6.9, and 7.7 μM, alongside EC_50_ values of 13.5, 14.0, and 5.0 μM, respectively [21,103]. Tanguticulines A (**199**) and E (**203**) were effective against H1N1, inhibiting the cytopathic effect with IC_50_ values of 2.9 and 2.4 μg/mL, respectively [85].

#### 3.5.2. Antibacterial Activity

Acoapetaludines D (**45**) and E (**46**) showed weak anti-*Helicobacter pylori* activity with minimum inhibitory concentrations (MICs) of 100 and 50 μg/mL, respectively [40]. Stylosines A (**157**) and B (**158**) exhibited antibacterial activity against *Staphylococcus aureus* with MIC of 2.00 and 32.00 μg/mL, respectively [74]. Koninginols A (**284**) and B (**285**) exhibited significant antibacterial activities (CMCC 63501) with MIC values of 10 and 2 μg/mL, respectively [102].

#### 3.5.3. Antiplasmodial Activity

2-O-cinnamoyl hetisine (**209**) showed antimalarial activity against the *Plasmodium falciparum* strains *Pf* INDO and *Pf* 3D7, with the IC_50_ values of 1.92 μM and 10.8 μM, respectively [88].

### 3.6. Other Activity

In addition to the widely recognized anti-inflammatory, analgesic, antitumor, and antimicrobial activities mentioned above, recent research reported over the past five years has uncovered several TeAs with other significant biological activities, including vascular relaxation activity, antifibrosis activity, and neuroprotective activities.

Alstochonines A (**1**) and B (**2**) showed moderate vasorelaxant activity with rates of 73.6 ± 2.8% and 95.4 ± 3.7%, making the first report of the vasorelaxant activity of monoterpene alkaloids [16]. Lomatogonin C(**26**) displayed immunosuppressive activity, further evidenced by the inhibition of T cell proliferation and secretion of its cytokine IFN–*γ* in T cells stimulated with the anti-CD3/CD28 antibody [26]. (±)-Caryopterisines A (**19**) and B (**20**) inhibited estrogen E2 biosynthesis in human ovarian granulosa-like KGN cells by 57.2% and 39.9% at a dose of 10 μM, respectively.

Caryopterisine C (**16**) showed potential antifibrotic activity without cytotoxicity by inhibiting collagen accumulation (IC_50_ = 14.26 ± 1.46 μM) in NIH3T3 cells (murine embryo fibroblasts). Further studies into proteins involved in transforming growth factor-*β*-activated signaling pathways revealed that caryopterisine C (**16**) reduced collagen accumulation by inhibiting ERK1/2, P38, and SMAD2/3 phosphorylation. Apetalrine B (**82**) showed neuroprotective activity, with a neuroprotective rate on H_2_O_2_-induced SH-SY5Y cell injury of 77.4%. Its neuroprotective effect is believed to be achieved by inhibiting apoptosis [55]. Uncinatine-A (**120**) showed significant acetylcholinesterase (AChE) inhibitory activity, with IC_50_ values of 207.73 ± 0.3 μM [66]. Anthoroidines G (**204**) and I (**206**) showed certain AChEI activity, with IC_50_ values of 6.3 ± 1.6 and 9.3 ± 3 μM, respectively [84].

## 4. Conclusions

This review summarizes the sources, chemical structures, and biological activities of 289 TeAs discovered between 2019 and 2024, including 26 monoterpenoid alkaloids, 6 sesquiterpenoid alkaloids, and 257 DAs. DAs are the most abundant class of terpenoid alkaloids widely distributed in *Aconitum* and *Delphinium*. Seven novel DAs (**283**–**289**) were obtained from *Spongia* sp., *Trichoderma koningii* A729, and *Forsythia suspensa* in the last five years, respectively. Monoterpene alkaloids are mainly distributed in *Apocynaceae*, *Scrophulariaceae*, and *Gentianaceae*, while sesquiterpene alkaloids, the least common class, are found in *Dendrobium*.

The majority of TeAs exhibit anti-inflammatory, antitumor, and antimicrobial properties. Among the terpenoid alkaloids discovered in the past five years, the analgesic activity is unique to diterpenoid alkaloids. Distinctively, the analgesic activity has been identified exclusively in diterpenoid alkaloids, with several DAs demonstrating analgesic effects in mice superior to standard drugs like aspirin and acetaminophen, including Episcopaline B, Pseudostapine C, and Austroyunnanine B. These findings support the potential for developing novel analgesics. Moreover, TeAs show promise as therapeutic agents for various cancers, exhibiting inhibitory effects on breast, intestinal, liver, lung, and cervical cancer cells in vitro. The efficacy of these compounds in vivo remains an area for future research. TeAs also hold potential as cardiovascular medications, exemplified by compounds like Smirnotine A, which has shown heart-protective activity.

While current research has revealed a broad spectrum of TeAs’ biological activities, most of these studies have been limited to in vitro cell viability assessments. There is a significant need for further in vivo pharmacological studies to understand the therapeutic potential of TeAs against various diseases comprehensively. This review has compiled the sources, structural characteristics, and biological activities of newly discovered compounds within the last five years, aiming to serve as a valuable resource for the continued exploration and application of terpenoid alkaloids in therapeutic contexts.

## Figures and Tables

**Table 7:** Names and plant sources of franchetine-type C19-DAs (**159**–**168**).

No.	Compound Name	Sources	Plant Parts	Ref.
**159**	Acotarine A	*Aconitum *taronense**	*root*	[56]
**160**	Acotarine B	*Aconitum *taronense**	*root*	[56]
**161**	Acotarine C	*Aconitum *taronense**	*root*	[56]
**162**	Acotarine D	*Aconitum *taronense**	*root*	[56]
**163**	Acotarine E	*Aconitum *taronense**	*root*	[56]
**164**	Flavumoline A	*Aconitum flavum* Hand.-Mazz	aerial parts	[76]
**165**	Flavumoline B	*Aconitum flavum* Hand.-Mazz	aerial parts	[76]
**166**	Flavumoline C	*Aconitum flavum* Hand.-Mazz	aerial parts	[76]
**167**	Flavumoline D	*Aconitum flavum* Hand.-Mazz	aerial parts	[76]
**168**	Rockidine C	*Aconitum genera*	*root*	[43]

**Table 8:** Names and plant sources of 7,17-*seco*-type C19-DAs (**169**–**175**).

No.	Compound Name	Sources	Plant Parts	Ref.
**169**	Grandifline B	*Delphinium grandiflorum*	aerial part	[73]
**170**	Aconicumine A	*Aconitum taipeicum* Hand.-Mazz.	root	[77]
**171**	Aconicumine B	*Aconitum taipeicum* Hand.-Mazz.	root	[77]
**172**	Aconicumine C	*Aconitum taipeicum* Hand.-Mazz.	root	[77]
**173**	Aconicumine D	*Aconitum taipeicum* Hand.-Mazz.	root	[77]
**174**	Brunodelphinine B	Delphinium brunonianum Royle	aerial part	[64]
**175**	Brunodelphinine D	Delphinium brunonianum Royle	aerial part	[64]

**Table 9:** Names and plant sources of rearranged C19-DAs (**176**–**182**).

No.	Compound Name	Sources	Plant Parts	Ref.
**176**	Nagarumine C	*Aconitum nagarum*	root	[46]
**177**	Acosinomonine A	*Aconitum sinomontanum*	root	[79]
**178**	Acosinomonine B	*Aconitum sinomontanum*	root	[79]
**179**	Grandifline A	*Delphinium grandiflorum* L.	whole herb	[73]
**180**	Episcopine A	*Aconitum episcopale*	root	[52]
**181**	Gyalanutine A	*Delphinium gyalanum* C. Marquand & Airy Shaw	whole plant	[78]
**182**	Episcopaline B	*Aconitum episcopale*	root	[50]

**Table 10:** Name and plant sources of atisine-type C20-DAs (**183**–**191**).

No.	Compound Name	Sources	Plant Parts	Ref.
**183**	Brunonianine A	*Delphinium brunonianum.*	whole plant	[80]
**184**	Brunonianine B	*Delphinium brunonianum.*	whole plant	[80]
**185**	Brunonianine C	*Delphinium brunonianum.*	whole plant	[80]
**186**	Delphatisine D	*Delphinium chrysotrichum*	aerial part	[69]
**187**	Barpubesine A	*Aconitum barbatum* var. *puberulum* Ledeb	whole plant	[37]
**188**	Barpubesine B	*Aconitum barbatum* var. *puberulum* Ledeb	whole plant	[37]
**189**	Barpubesine C	*Aconitum barbatum* var. *puberulum* Ledeb	whole plant	[37]
**190**	Forrestline F	*Delphinium forrestii* var. *viride*	whole plant	[53]
**191**	Brunodelphinine E	Delphinium brunonianum	aerial parts	[64]

**Table 11:** Names and plant sources of hetisine-type C20-DAs (**192**–**217**).

No.	Compound Name	Sources	Plant Parts	Ref.
**192**	Trichophorine A	*Delphinium trichophorum* Franch.	whole herb	[83]
**193**	Trichophorine B	*Delphinium trichophorum* Franch.	whole herb	[83]
**194**	Trichophorine C	*Delphinium trichophorum* Franch.	whole herb	[83]
**195**	Coreanine A	*Aconitum coreanum*	root	[87]
**196**	Coreanine B	*Aconitum coreanum*	root	[87]
**197**	Coreanine C	*Aconitum coreanum*	root	[87]
**198**	Coreanine D	*Aconitum coreanum*	root	[87]
**199**	Tanguticuline A	*Aconitum tanguticum* (Maxim.) Stapf	whole plant	[85]
**200**	Tanguticuline B	*Aconitum tanguticum* (Maxim.) Stapf	whole plant	[85]
**201**	Tanguticuline C	*Aconitum tanguticum* (Maxim.) Stapf	whole plant	[85]
**202**	Tanguticuline D	*Aconitum tanguticum* (Maxim.) Stapf	whole plant	[85]
**203**	Tanguticuline E	*Aconitum tanguticum* (Maxim.) Stapf	whole plant	[85]
**204**	Anthoroidine G	*Aconitum *anthoroideum** DC.	whole plant	[84]
**205**	Anthoroidine H	*Aconitum *anthoroideum** DC.	whole plant	[84]
**206**	Anthoroidine I	*Aconitum *anthoroideum** DC.	whole plant	[84]
**207**	Grandiflonine C	*Delphinium grandiflorum* L.	whole plant	[68]
**208**	Grandiflonine D	*Delphinium grandiflorum* L.	whole plant	[68]
**209**	2-*O*-cinnamoyl hetisine	*Aconitum *heterophyllum**	root	[88]
**210**	hetisane-15*β*-*O*-*β*-d-glucoside	*Aconitum carmichaelii* Debx.	root	[47]
**211**	Hydrodavisine	*Delphinium peregrinum* L. var. *eriocarpum Boiss*	aerial part	[59]
**212**	Pachycentine	*Delphinium pachycentrum* Hemsl	whole herb	[86]
**213**	Anthoroidine A	*Aconitum *anthoroideum** DC.	whole plant	[84]
**214**	Ajacisine H	*Delphinium ajacis*	seed	[70]
**215**	Tanguticuline F	*Aconitum tanguticum* (Maxim.) Stapf	whole plant	[85]
**216**	Tanguticuline G	*Aconitum tanguticum* (Maxim.) Stapf	whole plant	[85]
**217**	Gyalanunine B	*Delphinium gyalanum* C. Marquand & Airy Shaw	whole plant	[89]

**Table 12:** Names and plant sources of hetidine-type C20-DAs (**218**–**228**).

No.	Compound Name	Sources	Plant Parts	Ref.
**218**	15-epinaviculine B	*Delphinium oreophilum*	aerial part	[81]
**219**	Tangutidine D	*Aconitum tanguticum* (Maxim.) Stapf	whole plant	[92]
**220**	Tangutidine E	*Aconitum tanguticum* (Maxim.) Stapf	whole plant	[92]
**221**	Paradoxine	*Delphinium paradoxum* Bunge	aerial part	[91]
**222**	Tangutidine A	*Aconitum tanguticum*	whole plant	[90]
**223**	Tangutidine B	*Aconitum tanguticum*	whole plant	[90]
**224**	Tangutidine C	*Aconitum tanguticum*	whole plant	[90]
**225**	Anthoroidine F	*Aconitum *anthoroideum** DC.	whole plant	[84]
**226**	Sczukiniline A	*Aconitum sczukinii* Turcz	root	[65]
**227**	Sczukiniline B	*Aconitum sczukinii* Turcz	root	[65]
**228**	Sczukiniline C	*Aconitum sczukinii* Turcz	root	[65]

**Table 13:** Names and plant sources of denudatine-type C20-DAs (**229**–**241**).

No.	Compound Name	Sources	Plant Parts	Ref.
**229**	Kirisine F	*Aconitum kirinense* Nakai	root	[35]
**230**	Kirisine G	*Aconitum kirinense* Nakai	root	[35]
**231**	Kirisine H	*Aconitum kirinense* Nakai	root	[35]
**232**	Kirisine I	*Aconitum kirinense* Nakai	root	[35]
**233**	Kirisine J	*Aconitum kirinense* Nakai	root	[35]
**234**	Kirisine K	*Aconitum kirinense* Nakai	root	[35]
**235**	Kirisine L	*Aconitum kirinense* Nakai	root	[35]
**236**	Barpubesine D	*Aconitum *barbatum** var. *puberulum*	whole plant	[37]
**237**	Aconicarnine C	*Aconitum carmichaelii*	lateral root	[93]
**238**	Aconicarnine A	*Aconitum carmichaelii*	lateral root	[93]
**239**	Aconicarnine B	*Aconitum carmichaelii*	lateral root	[93]
**240**	Aconicarnine D	*Aconitum carmichaelii*	lateral root	[93]
**241**	Aconicarnine E	*Aconitum carmichaelii*	lateral root	[93]

**Table 14:** Names and plant sources of napelline-type C20-DAs (**242**–**249**).

No.	Compound Name	Sources	Plant Parts	Ref.
**242**	Kirisine M	*Aconitum kirinense* Nakai	root	[35]
**243**	Kirisine N	*Aconitum kirinense* Nakai	root	[35]
**244**	Kirisine O	*Aconitum kirinense* Nakai	root	[35]
**245**	12-epi-aconicarmichinium A	*Aconitum pendulum* Busch	root	[96]
**246**	Napelline **C**	*Aconiti kusnezoffii Radix*	root	[94]
**247**	Napelline D	*Aconiti kusnezoffii Radix*	root	[94]
**248**	Napelline E	*Aconiti kusnezoffii Radix*	root	[94]
**249**	Chuanfusulfonine A	*Aconitum carmichaelii*	lateral root	[95]

**Table 15:** Names and plant sources of vakognavine-type C20-DAs (**250**–**255**).

No.	Compound name	Sources	Plant parts	Ref.
**250**	Rockisine A	*Aconitum genera*	root	[43]
**251**	Umbrodine A	*Delphinium umbrosum* Hand.-Mazz.	whole plant	[97]
**252**	Kingiadine	*Delphinium kingianum* Bruhl. ex Huth.	whole plant	[97]
**253**	Gyalanunine A	*Delphinium *gyalanum** C. Marquand & Airy Shaw	whole plant	[89]
**254**	Grandiflonine A	*Delphinium grandiflorum* L.	whole plant	[68]
**255**	Grandiflonine B	*Delphinium grandiflorum* L.	whole plant	[68]

**Table 16:** Names and plant sources of rearranged C20-DAs (**256**–**266**).

No.	Compound Name	Sources	Plant Parts	Ref.
**256**	Anthoroidine C	*Aconitum *anthoroideum DC.**	whole plant	[84]
**257**	Anthoroidine D	*Aconitum *anthoroideum DC.**	whole plant	[84]
**258**	Anthoroidine E	*Aconitum *anthoroideum DC.**	whole plant	[84]
**259**	Aconicatisulfonine A	*Aconitum carmichaelii*	lateral root	[98]
**260**	Aconicatisulfonine B	*Aconitum carmichaelii*	lateral root	[98]
**261**	Aconapelsulfonine A	*Aconitum carmichaelii*	lateral root	[99]
**262**	Aconapelsulfonine B	*Aconitum carmichaelii*	lateral root	[99]
**263**	Aconicarmisulfonine B	*Aconitum carmichaelii*	lateral root	[95]
**264**	Aconicarmisulfonine C	*Aconitum carmichaelii*	lateral root	[95]
**265**	Barpuberudine	*Aconitum* barbatum var. *puberulum* Ledeb	whole plant	[37]
**266**	Acoapetaludine A	*Aconitum apetalum* (Huth) B.Fedtsch	whole plant	[40]

**Table 17:** Names and plant sources of Bis-DAs (**267**–**280**).

No.	Compound Name	Types	Sources	Plant Parts	Ref.
**267**	Weisaconitine E	denudatine-atisine	*Aconitum weixiense*	root	[100]
**268**	Weisaconitine F	denudatine-atisine	*Aconitum weixiense*	root	[100]
**269**	Tangirine A	heteratisine-hetidine	*Aconitum tanguticum* (Maxim.) Stapf.	whole plant	[92]
**270**	Tanguticinine A	hetidine-hetisine	*Aconitum tanguticum* (Maxim.) Stapf.	whole plant	[92]
**271**	Tanguticinine B	hetidine-hetisine	*Aconitum tanguticum* (Maxim.) Stapf.	whole plant	[92]
**272**	Tanguticinine C	hetidine-hetisine	*Aconitum tanguticum* (Maxim.) Stapf.	whole plant	[92]
**273**	Tanguticinine D	hetidine-hetisine	*Aconitum tanguticum* (Maxim.) Stapf.	whole plant	[92]
**274**	Tanguticinine E	hetidine-hetisine	*Aconitum tanguticum* (Maxim.) Stapf.	whole plant	[92]
**275**	Tanguticinine F	hetidine-hetisine	*Aconitum tanguticum* (Maxim.) Stapf.	whole plant	[92]
**276**	Tanguticinine G	hetidine-atisine	*Aconitum tanguticum* (Maxim.) Stapf.	whole plant	[92]
**277**	Anthoroidine B	hetidine-rearranged hetisine	*Aconitum anthoroideum* DC.	whole plant	[84]
**278**	N-oxide anthoroidine B	hetidine-rearranged hetisine	*Aconitum tanguticum* (Maxim.) Stapf.	whole plant	[92]
**279**	5-deoxyanthoridine B	hetidine-rearranged hetisine	*Aconitum tanguticum* (Maxim.) Stapf.	whole plant	[92]
**280**	N-oxide 5-deoxyanthoroidine B	hetidine-rearranged hetisine	*Aconitum tanguticum* (Maxim.) Stapf.	whole plant	[92]

**Table 18:** Names and plant sources of other DAs (**281**–**289**).

No.	Compound Name	Sources	Ref.
**281**	Ceylonamide G	*Spongia* sp.	[101]
**282**	Ceylonamide H	*Spongia* sp.	[101]
**283**	Ceylonamide I	*Spongia* sp.	[101]
**284**	Koninginol A	*Trichoderma koningiopsis* A729	[102]
**285**	Koninginol B	*Trichoderma koningiopsis* A729	[102]
**286**	Forsyqinlingine A	*Forsythia suspensa*	[103]
**287**	Forsyqinlingine B	*Forsythia suspensa*	[103]
**288**	Aconidenusulfonine A	*Aconitum carmichaelii*	[104]
**289**	12,16-secoaconidenusulfonine A	*Aconitum carmichaelii*	[104]

**Table 19:** Biological activity of terpenoid alkaloids.

TeAs Types	Activities	Research Method	Possible Mechanism	Ref.
**Monoterpenoid Alkaloids**				
Alstochonine A (**1**)	Vasorelaxant	In vitro	Vasorelaxant activity against phenylephrine-induced contraction of rat mesenteric arteries with rates of 73.6 ± 2.8% and 95.4 ± 3.7% (IC_50_ = 93.30 ± 10.81, 60.56 ± 3.66 μM)	[16]
Alstochonine B (**2**)		In vitro		[16]
Delavatine C (**9**)	Anti-inflammatory	In vitro	Inhibition of NO production in LPS-stimulated BV2 cells (IC_50_ = 25.62, 17.29 μM)	[18]
Delavatine E (**11**)		In vitro		[18]
Forsyqinlingine C (**14**)	Anti-inflammatory and antiviral	In vitro	Anti-inflammatory activities by inhibiting the release of β-glucuronidase in PMNs with inhibition rates of 45.2% and 40.1%, and antiviral activities against H1N1 virus (IC_50_ = 11.9, 15.1 μM) and RSV (EC_50_ = 13.5, 14.0 μM)	[21]
Forsyqinlingine D (**15**)		In vitro		[21]
Incarvine G (**12**)	Antitumor	In vitro	Cytotoxicity (IC_50_ = 60.29 μM) against MDA-MB-231 cells and inhibiting actin cytoskeleton formation	[19]
(±)-Caryopterisine A (**19**)		In vitro	Reduction of Kyn biosynthesis in HeLa cells by inhibiting IDO at 10 μM with inhibition ratios of 25.7% and 29.8%, respectively	[22]
(±)-Caryopterisine B (**20**)		In vitro		[22]
Caryopterisine C (**16**)	Antifibrotic	In vitro	Inhibition of collagen accumulation (IC_50_ = 14.26 ± 1.46 μM) in NIH3T3 cells and phosphorylation of ERK1/2, P38, and SMAD2/3	[23]
Lomatogonin C (**26**)	Immunosuppressive	In vitro	Inhibition of T cell proliferation (21.62 ± 3.06%) and IFN–γ secretion (37.59 ± 5.41%) at 20 μM	[26]
**Sesquiterpene alkaloids**				
Commipholactam A (**27**)	Antitumor	In vitro	Cytotoxicity against HepG2 (IC_50_ = 21.73 ± 2.86 μM) and A549 (IC_50_ = 128.50 ± 17.06 μM) cells	[28]
**Diterpenoid alkaloids**				
Geordine (**103**)	Anti-inflammatory	In vitro	Inhibition of NO production (29.75%) in LPS-induced RAW264.7 cells at 50 μM	[61]
Stylosine A (**157**)		In vitro	Inhibition of production of IL-1β, COX-2, and TNF-α in LPS-induced RAW264.7 cells in a dose-dependent manner	[74]
Ajacisine F (**121**)		In vitro	Inhibition of NO production in LPS-induced BV-2 cells with inhibition rates of 80% at 50 μM	[70]
Ajacisine G (**123**)		In vitro		[70]
Ajacisine H (**214**)		In vitro		[70]
Kamaonensine B (**148**)		In vitro	Inhibition of NO production in LPS-stimulated RAW264.7 cells (IC_50_ = 2.7 ± 0.5 and 0.9 ± 0.2 μM) and might be mediated by the regulation of some related proteins in the MAPK signaling pathways	[72]
Kamaonensine F (**153**)		In vitro		[72]
Aconicumine A (**170**)		In vitro	Inhibition of LPS-activated NO production in RAW264.7 cells (IC_50_ = 19.7 ± 1.1 μM)	[77]
Forrestline F (**190**)		In vitro	Inhibition of NO production in RAW264.7 cells (IC_50_ = 9.57 ± 1.34 μM) through inhibiting NF-*κ*B, MAPK, and Nrf2 signaling pathways	[53]
Anthoroidine B (**277**)		In vitro	Inhibition of the production of NO and TNF-*α* in LPS-stimulated RAW264.7 cells, with IC_50_ values of 357.68 and 67.56 μM	[84]
Forsyqinlingine A (**286**)	Anti-inflammatory and antiviral	In vitro	Anti-inflammatory activities by inhibiting the release of β-glucuronidase in PMNs with inhibition rates of 56.7 and 58.6%, and antiviral activities against H1N1 virus (IC_50_ = 6.9 and 7.7 μM) and RSV (EC_50_ = 5.0 and 4.8 μM)	[103]
Forsyqinlingine B (**287**)		In vitro		[103]
Pseudostapine C (**89**)	Analgesic	In vivo	Reduction of acetic acid-induced abdominal contractions in mice in a dose-related manner, with the ID_50_ values of 66.1, 60.3, 48.0, and 55.0 μmol/kg, respectively	[51]
Austroyunnanine B (**91**)		In vivo		[45]
Episcopine A (**180**)		In vivo		[52]
Episcopaline B (**182**)		In vivo		[50]
Nagarumine C (**176**)		In vivo	Inhibition of acetic acid-induced writhing in mice (ED_50_ = 76.0 μmol/kg)	[46]
Acosinomonine B (**178**)		In vitro	Strong inhibitory effect on the activation of the TRPV1 channel in HEK-293 cells mediated by capsaicin, with an inhibition rate of 31.78% at 10 μM	[79]
Aconicatisulfonine A (**259**)		In vivo	Reduction in acetic acid-induced writhing in mice by 43.2%, 64.7%, 63.6%, and 19.3% at 0.3 mg/kg, respectively	[98]
Aconicatisulfonine B (**260**)		In vivo		[98]
Aconapelsulfonine A (**261**)		In vivo		[99]
Aconapelsulfonine B (**262**)		In vivo		[99]
Aconicarmisulfonine B (**263**)		In vivo	Reduction in acetic acid-induced writhing in mice by 31.26%, 26.84%, and 43.8% at 1.0 mg/kg (i.p.)	[95]
Aconicarmisulfonine C (**264**)		In vivo		[95]
Aconicarnine E (**241**)		In vivo		[93]
Aconidenusulfonine A (**288**)		In vivo	Reduction in acetic acid-induced writhing in mice by 26.35% at 2.0 mg/kg (i.p.)	[104]
Lipojesaconitine (**98**)	Antitumor	In vitro	Cytotoxicity against A549, MDA-MB-231, MCF-7, and KB with IC50 values ranging from 6.0 to 7.3 μM	[57]
8-O-ethyl-benzoyldeoxyaconine (**107**)		In vitro	Anticancer activity against A549 (IC_50_ = 12.58 ± 1.82 μM) and H460 (IC_50_ = 12.76 ± 2.10 μM) cells	[47]
Brunonianine B (**182**)		In vitro	Cytotoxicity on Caco-2 (IC_50_ = 3.14 ± 0.37 μM) and Skov-3 (IC_50_ = 2.20 ± 0.21 μM) cells and activation of the Bax/Bcl-2/caspase-3 signaling pathway	[80]
Brunonianine C (**183**)		In vitro	Cytotoxicity on Caco-2 (IC_50_ = 2.41 ± 0.35 μM) and Skov-3 (IC_50_ = 6.88 ± 0.81 μM) cells	[80]
Ceylonamide G (**281**)		In vitro	Cytotoxic to DU145 cells (IC_50_ = 6.9 μM, MEC = 10 μM)	[101]
Smirnotine A (**94**)	Cardioprotective	In vivo	Some preventive effects on aconitine-induced arrhythmia in mice	[58]
Gyalanunine A (**253**)		In vitro	Significant cardiotonic activity after perfusion in frog hearts and could be related to the *β* receptor	[89]
Tanguticuline A (**199**)	Antiviral	In vitro	Inhibition of the cytopathic effect against H1N1 with IC_50_ values of 2.9 and 2.4 μg/mL, respectively	[85]
Tanguticuline E (**203**)		In vitro		[85]
Acoapetaludine D (**45**)	Antibacterial	In vitro	Anti-*Helicobacter pylori* (MIC = 100 μg/mL)	[40]
Acoapetaludine E (**46**)		In vitro	Anti-*Helicobacter pylori* (MIC = 50 μg/mL)	[40]
Stylosine A (**157**)		In vitro	Anti-*Staphylococcus aureus* (MIC = 2.00 μg/mL)	[74]
Stylosine B (**158**)		In vitro	Anti-*Staphylococcus aureus* (MIC = 32.00 μg/mL)	[74]
Koninginol A (**284**)		In vitro	Anti-*Bacillus subtilis* (MIC = 10.00 μg/mL)	[102]
Koninginol B (**285**)		In vitro	Anti-*Bacillus subtilis* (MIC = 2.00 μg/mL)	[102]
2-O-cinnamoyl hetisine (**209**)	Antiplasmodial	In vitro	Anti-*Plasmodium falciparum* strains *Pf* INDO (IC_50_ = 1.92 μM) and the *Pf* 3D7 (IC_50_ = 10.8 μM)	[88]
Apetalrine B (**82**)	Neuroprotective	In vitro	Neuroprotective activity (77.4%) on H_2_O_2_-induced SH-SY5Y cell	[55]
Uncinatine-A (**120**)	AChEI activity	In vitro	Acetyl-cholinesterase inhibitory activity (IC_50_ = 207.73 ± 0.3 μM	[66]
Anthoroidine G (**204**)		In vitro	Acetyl-cholinesterase inhibitory activity (IC_50_ = 6.3 ± 1.6 μM)	[84]
Anthoroidine I (**206**)		In vitro	Acetyl-cholinesterase inhibitory activity (IC_50_ = 9.3 ± 3 μM)	[84]

## Data Availability

Not applicable.

## Abbreviations

TeAs	Terpenoid alkaloids
MEP	Methylerythritol phosphate
NMR	Nuclear magnetic resonance
HR-ESI	High-resolution mass spectrometry
ECD	Electron capture detector
DAs	Diterpenoid alkaloids
TNF-α	Tumor necrosis factor-α
IL-1*β*	Interleukin-1*β*
NF-κB	Nuclear factor-*κ*B
TRPV1	Transient receptor vanilloid 1
MEC	Minimum effective concentration
HEK-293	Human Embryonic Kidney 293 Cells
LPS	Lipopolysaccharide
COX-2	Cyclooxygenase-2
PMNs	Polymorphonuclear leukocytes
PAF	Platelet-activating factor
MAPK	Mitogen-activated protein kinase
AChE	Acetylcholinesterase
MICs	Minimum inhibitory concentrations
IC_50_	Half maximal inhibitory concentration
ID_50_	Half infectious dose
ERK1/2	Extracellular signal-regulated kinase 1/2
ROS	Reactive oxygen species
Nrf2	NF-E2-related factor 2
Kyn	Kynurenine
IDO	Indoleamine 2,3-dioxygenase
P-gp	P-glycoprotein
HCPT	Hydroxy camptothecin
RSV	Respiratory syncytial virus
H1N1	Influenza A virus
IFN–γ	Interferon-γ
Bax	Bcl-2-associated X protein
Bcl-2	B-cell lymphoma-2
P38	p38 mitogen-activated protein kinase (MAPK)
SMAD 2/3	Mothers against decapentaplegic homolog 2/3
CMCC 63501	*Bacillus subtilis*
Me	Methyl
Et	Ethylic
Ibut	Isobutyryl
Mb	2-metylbutyryl
Bz	Benzoyl
As	Anisoyl
Vr	Veratroyl
Cinn	Cinnamoyl
Ant	Anthranoyl
Glc	Glucose
Ac	Acetyl
pHb	p-hydroxybenzoyl
lipo	Stearoyl,oleoyl,linolenoyl,linoleoyl,palmitoly
